# Supplementary material for: Constructing a T-Cell Receptor-Related Gene Signature for Prognostic Stratification and Therapeutic Guidance in Head and Neck Squamous Cell Carcinoma
Source: Cancers (Basel). 2023 Nov 21;15(23):5495. doi: 10.3390/cancers15235495 (PMC10705130; doi:10.3390/cancers15235495)
Supplement: Supplementary file 1 [file cancers-15-05495-s001.zip › cancers-2688674-supplementary.pdf]

Constructing a T-Cell Receptor-Related Gene Signature for Prognostic Stratification and Therapeutic Guidance in Head and Neck Squamous Cell Carcinoma

Ye Lu 1,†, Zizhao Mai 1,†, Jiarong Zheng 2, Pei Lin 1, Yunfan Lin 1, Li Cui 1,* and Xinyuan Zhao 1,*

[1] Stomatological Hospital, School of Stomatology, Southern Medical University, Guangzhou 510280, China

[2] Department of Dentistry, the First Affiliated Hospital, Sun Yat-Sen University, Guangzhou 510080, China.

† These authors contributed equally to this work.

* Correspondence

Xinyuan Zhao, Email: zhaoxinyuan1989@smu.edu.cn

Li Cui, Email: licui@smu.edu.cn

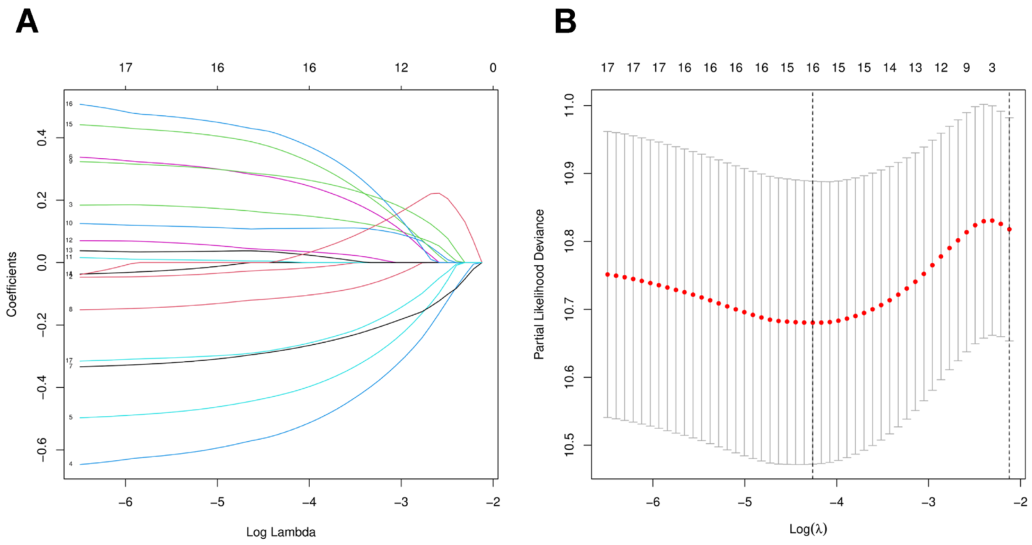

Supplementary Figure S1. Development of a TCRRG-based prognostic signature via LASSO regression analysis. (A) LASSO coefficient profiles of the 17 genes in the training cohort. (B) Selection of the optimal parameter (lambda) in the LASSO model.

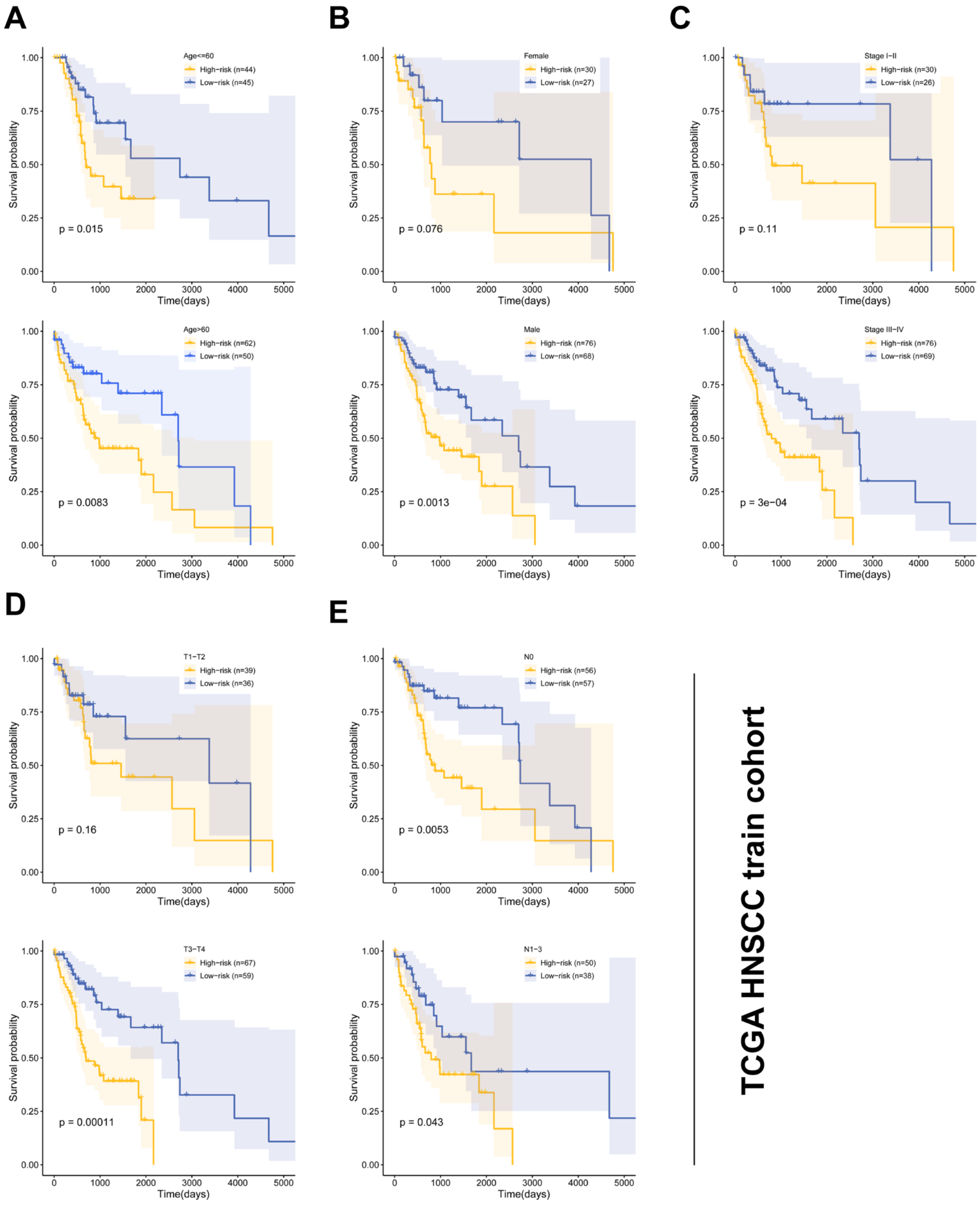

Supplementary Figure S2. Analysis of survival difference in high- and low-risk groups as stratified by clinicopathological parameters within the TCGA HNSCC training Cohort. (A-E) Demonstration of different OS rates between high and low-risk groups, further classified by age, gender, clinical stage, T stage, and N stage.

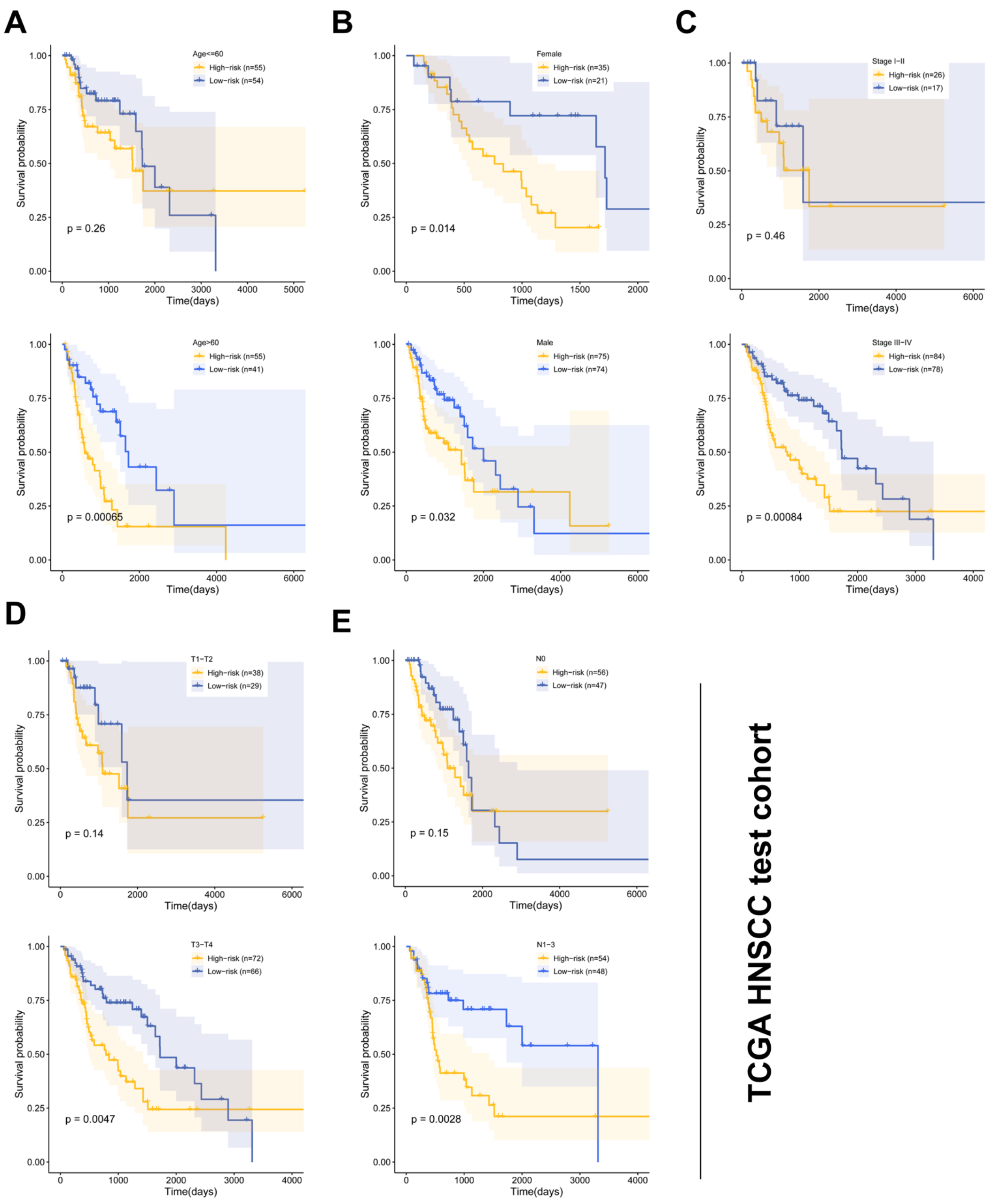

Supplementary Figure S3. Survival differences between high- and low-risk groups stratified by clinicopathological parameters in the TCGA HNSCC test cohort. (A-E) Illustration of differences in OS between high and low-risk groups, stratified by age, gender, clinical stage, T stage, and N stage respectively.

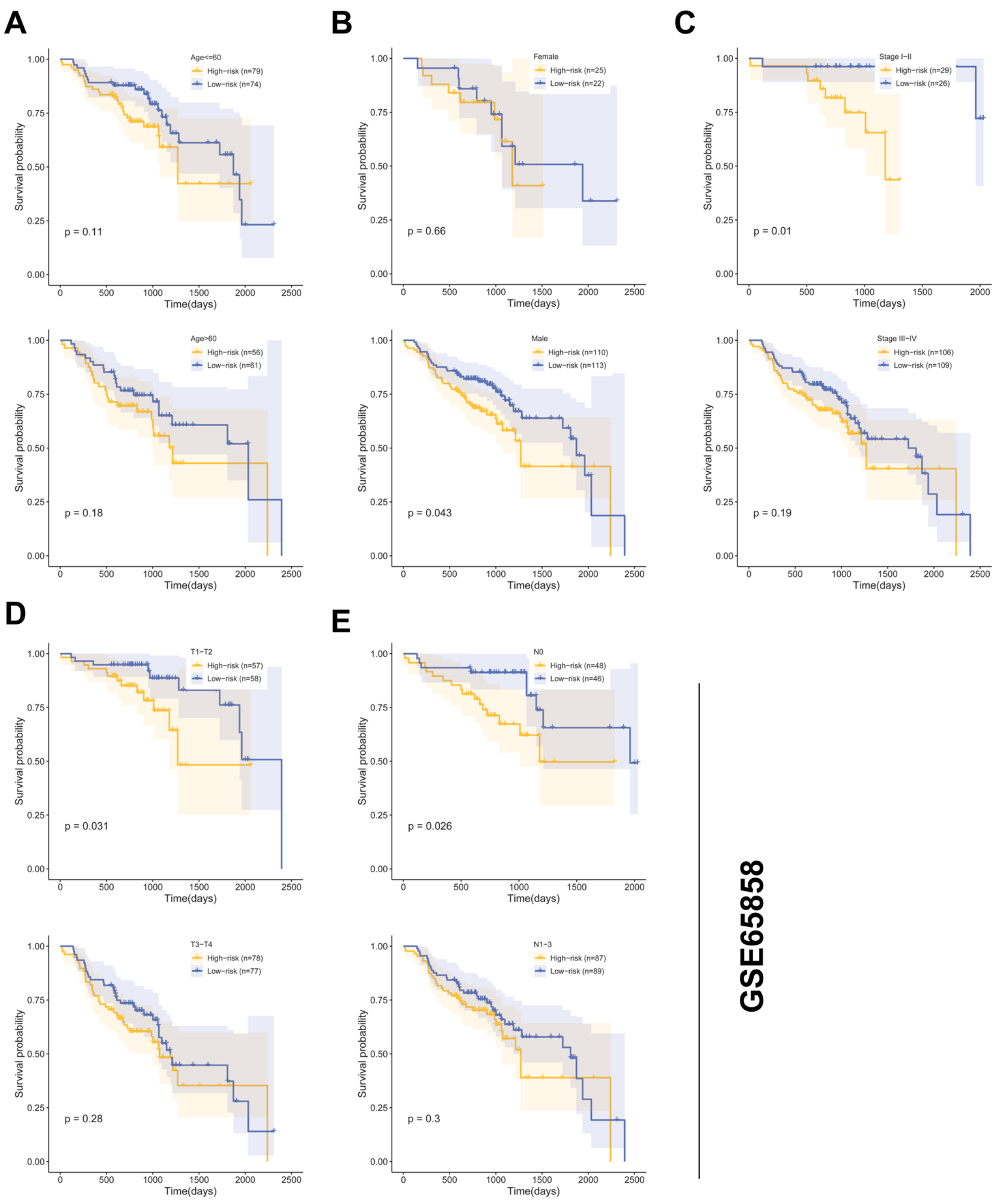

Supplementary Figure S4. Survival differences between high- and low-risk groups stratified by clinicopathological parameters in the GSE65858 cohort. (A-E) Presentation of differences in OS between high and low-risk groups, stratified by the indicated clinicopathological parameters.

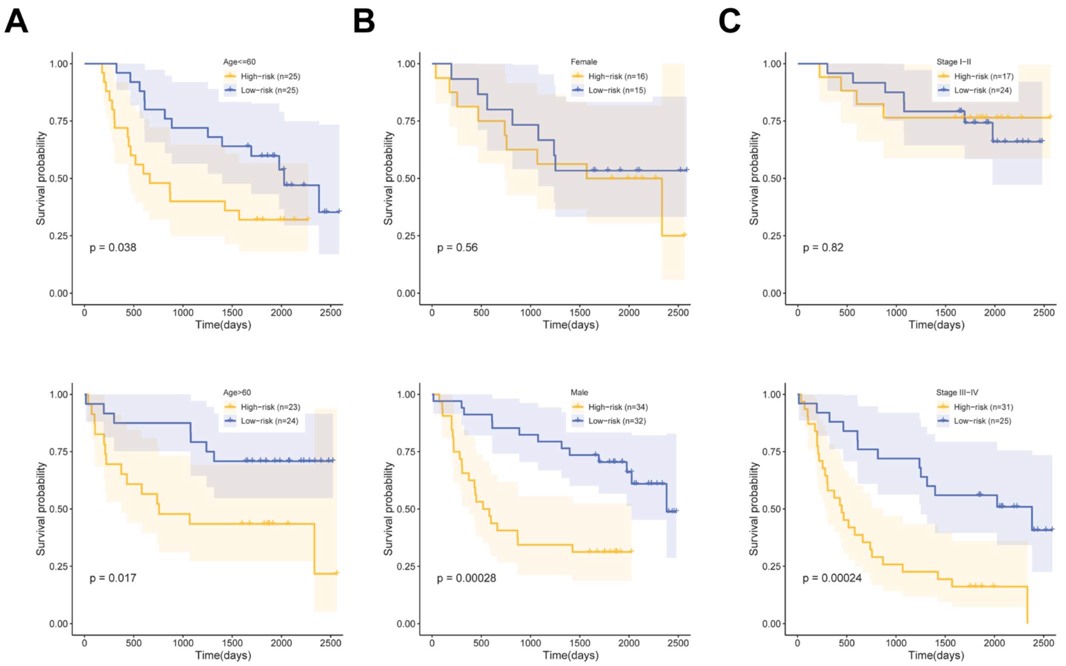

GSE41613

Supplementary Figure S5. Survival differences between high- and low-risk groups stratified by clinicopathological parameters in the GSE41613 cohort. (A-C) Exhibition of differences in OS between high and low-risk groups, stratified by age, gender and clinical stage respectively.

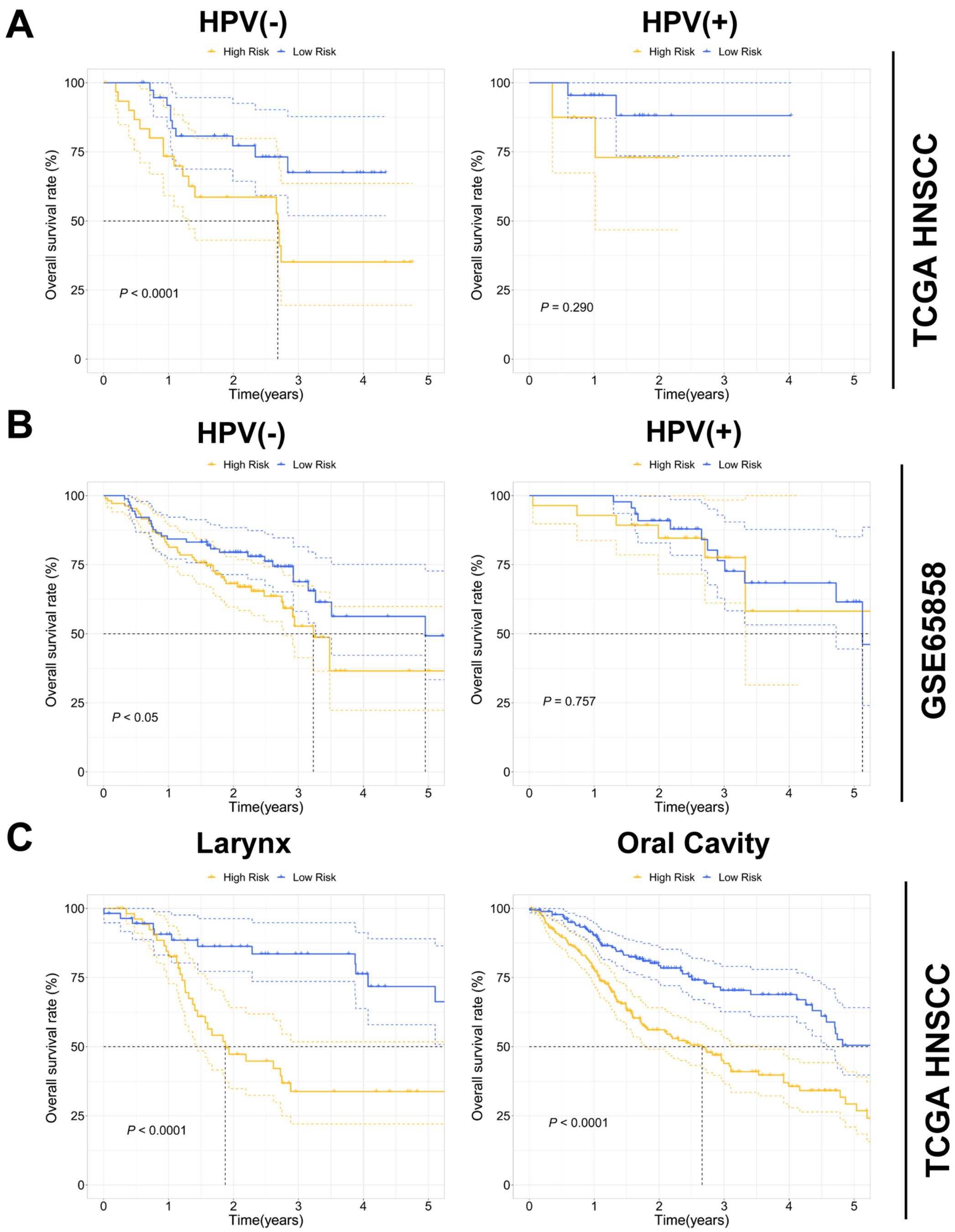

Supplementary Figure S6. Survival differences between high- and low-risk groups stratified by HPV status and primary tumor site. (A-B) Exhibition of differences in OS between high and low-risk groups, stratified by HPV status. (C) Presentation of differences in OS between high and low-risk groups, stratified by the different primary tumor site.

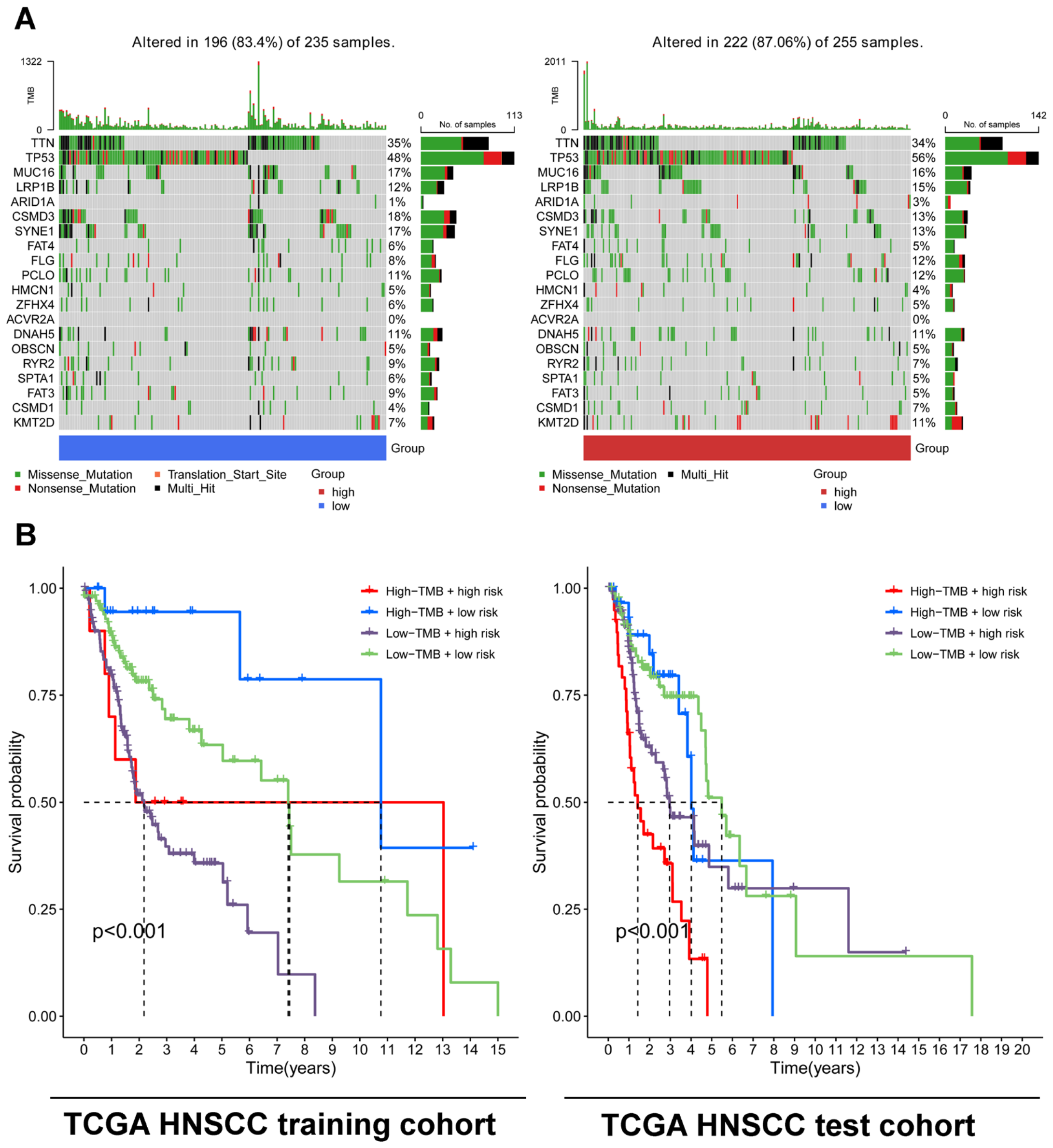

Supplementary Figure S7. Genomic alterations in low- and high-risk HNSCC groups and their impact on survival rates. (A) Waterfall plot of somatic mutation features in the high and low-risk groups. (B) Differences in OS in the indicated groups within the TCGA HNSCC training and test cohorts.

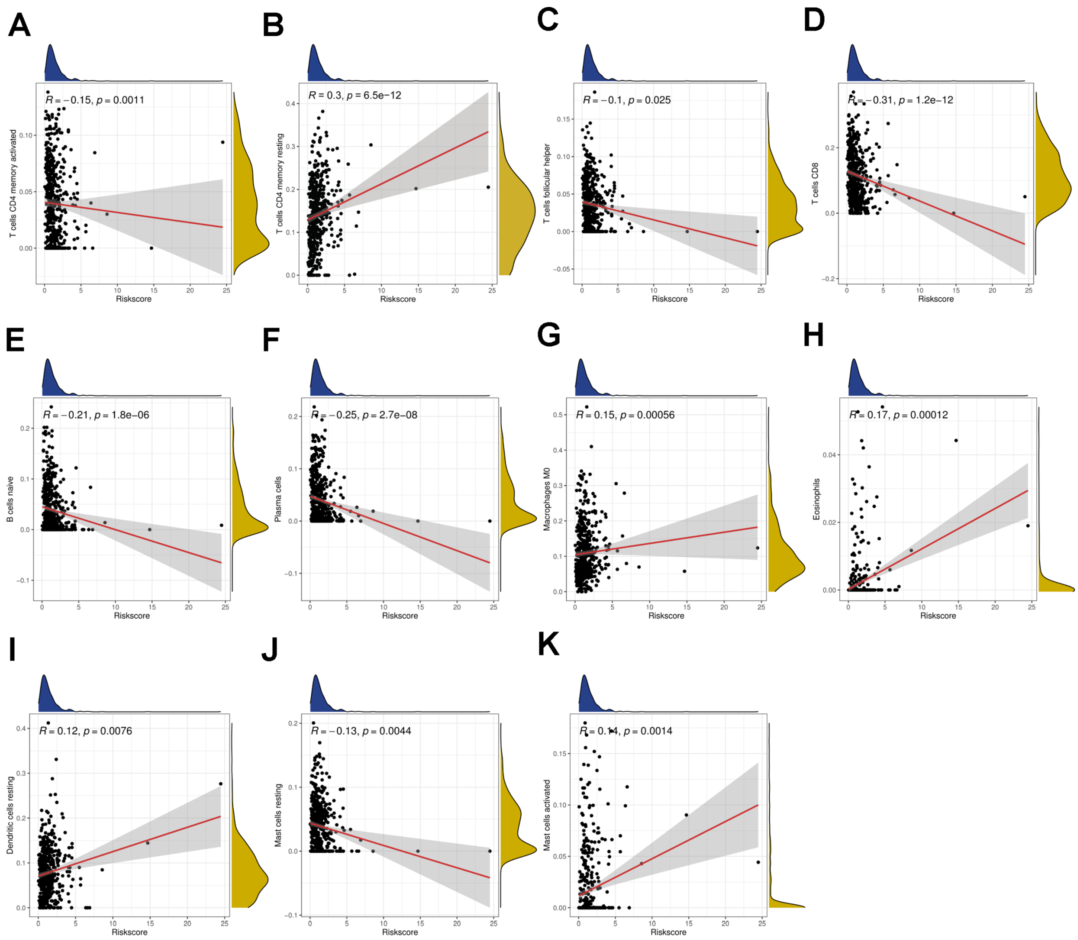

Supplementary Figure S8. Interplay between TCRRG-based risk score and immune cell infiltration in HNSCC. (A-K) Visualization of Pearson correlation analysis, outlining the relationship between the risk score and various immune cell types within the TCGA HNSCC cohort.

Supplementary Table S1. The riskscores of HNSCC patients in the TCGA HNSCC train cohort.

ID	futime	fustat	MAP2K7	MAPK3	MAPK9	ORAI1	PSMA1	UBB	ZAP70	Riskscore	Risk
TCGA-BA-4074	1.265	1	1.70715	3.1034	2.26763	4.01435	6.00891	7.02234	0.75382	8.57836	high
TCGA-BA-4076	1.13583	1	2.92782	4.77363	2.41628	3.20904	5.33255	8.24961	0.41546	3.60191	high
TCGA-BA-4078	0.75583	1	3.47993	3.29929	2.01272	3.91903	4.1413	8.37221	0.89443	1.43209	high
TCGA-BA-5152	3.52583	0	1.99998	4.16355	2.70251	4.9824	4.33222	8.32553	0.89848	2.91806	high
TCGA-BA-5556	1.985	0	2.95311	4.31714	2.07862	3.57904	4.47923	7.67325	1.54077	0.93982	low
TCGA-BA-5558	5.46167	0	3.18282	4.55656	1.90366	4.01579	4.31397	8.23858	0.71162	0.96802	low
TCGA-BA-6869	1.76333	0	3.18181	3.92751	2.1943	4.02318	4.57953	7.36832	1.91428	0.66085	low
TCGA-BA-6870	1.235	1	2.8792	4.16387	2.059	3.14653	4.24448	7.9894	0.84692	1.79068	high
TCGA-BA-6871	0.29583	1	3.10753	3.59696	2.34626	4.41807	4.46803	8.10191	1.34022	1.35692	high
TCGA-BA-6873	0.33417	0	3.26067	3.85888	2.22166	3.67985	4.48828	7.89831	0.96927	1.43468	high
TCGA-BA-A4IF	2.45	0	3.50958	4.27311	2.45932	3.66427	4.60315	6.2232	1.45885	0.42398	low
TCGA-BA-A4IG	2.34083	0	3.6293	3.96757	2.76329	4.05707	4.50787	7.66904	1.37236	0.92682	low
TCGA-BA-A4IH	1.7025	0	3.52521	3.80821	2.72594	3.37259	4.8754	6.18981	1.50995	0.74977	low
TCGA-BA-A6DA	0.96083	0	2.5953	4.99981	1.01165	3.39372	4.69138	7.51338	0.64992	0.78222	low
TCGA-BA-A6DG	0.18917	1	2.96096	3.85346	2.08382	3.71416	4.34526	7.77471	1.16738	1.30572	high
TCGA-BA-A6DI	0.92	1	3.06158	3.97711	1.82285	3.02474	4.4124	7.94724	0.48639	1.96948	high
TCGA-BA-A6DJ	1.11417	1	3.2401	4.24804	2.16496	4.28821	4.94416	7.82551	1.1906	1.00154	low
TCGA-BA-A8YP	1.36583	0	3.55102	4.35785	2.13307	3.61772	4.90623	7.87849	0.3792	1.41333	high
TCGA-BB-4225	0.4	0	3.32504	3.78622	2.34002	4.99021	4.94633	9.20694	2.7396	0.94819	low
TCGA-BB-4228	1.53	0	3.26976	4.1991	2.21888	4.25224	4.36023	8.83833	1.45886	1.14518	high
TCGA-BB-A5HZ	2.26417	0	3.78181	4.33425	1.59377	3.74873	4.3264	4.58808	1.72717	0.06946	low
TCGA-C9-A480	1.05667	0	3.03051	4.85554	2.12618	3.50199	4.35682	7.64023	0.72736	0.98225	low
TCGA-CN-4725	3.1675	0	2.92982	4.26926	1.61881	4.52211	4.22026	6.91526	0.26448	0.57888	low
TCGA-CN-4726	0.38833	1	2.65451	5.11263	2.51364	2.92964	4.04433	8.41928	0.20862	2.83414	high
TCGA-CN-4728	4.72	0	2.82945	3.92752	2.19857	3.51888	3.93813	8.17378	0.92271	1.7854	high
TCGA-CN-4730	2.23667	0	3.07496	4.55776	1.50606	4.2275	4.73856	7.54712	0.44963	0.75782	low
TCGA-CN-4733	4.34167	0	3.01812	3.96188	2.38673	4.10831	4.2498	8.22913	2.24983	0.87793	low
TCGA-CN-4734	4.62667	0	2.62204	4.03013	1.8507	3.23173	4.01985	7.61408	1.09935	1.31452	high
TCGA-CN-4735	4.755	0	2.62813	4.58831	2.69039	4.30966	4.09263	7.63828	1.10606	1.12581	high
TCGA-CN-4736	1.08167	1	2.6448	4.14518	2.43334	3.64336	3.9899	8.13498	1.21447	1.76281	high
TCGA-CN-4739	3.81667	1	3.44794	3.85366	2.1504	4.59431	3.57645	7.73031	2.48588	0.25075	low
TCGA-CN-4742	1.08667	1	2.46963	4.73251	2.07394	3.80262	4.5845	6.95928	0.30193	1.32106	high
TCGA-CN-5355	3.49833	0	3.22138	3.94368	1.99415	3.98338	4.45883	7.7874	0.6246	1.21236	high
TCGA-CN-5356	3.8575	0	2.98115	3.8816	2.13896	3.75474	3.22193	5.59882	0.93966	0.29308	low
TCGA-CN-5358	0.71417	1	3.17022	4.3186	2.14813	3.66351	4.10404	7.51958	2.1855	0.46567	low
TCGA-CN-5359	1.0325	1	2.80037	4.50369	2.54069	3.62909	3.82928	7.99099	2.01607	0.84382	low
TCGA-CN-5360	5.9375	0	3.52781	4.19729	2.18401	4.38806	4.18021	7.53348	2.32613	0.28881	low
TCGA-CN-5363	0.6925	1	2.9346	3.86367	2.91057	3.53839	4.17127	8.22921	0.69676	3.26725	high
TCGA-CN-5370	0.70917	1	2.77297	4.14286	2.68747	3.84135	4.37487	8.04072	1.15821	2.0375	high
TCGA-CN-6010	4.16917	0	3.35472	3.80851	2.13094	4.10487	4.39209	7.53529	0.71072	1.00101	low
TCGA-CN-6011	2.55417	0	3.7748	3.51762	2.44334	3.15494	3.54599	7.53536	0.60782	1.02747	low
TCGA-CN-6012	3.99667	0	3.22144	4.08654	1.9056	4.38098	3.61587	7.60716	1.33512	0.40684	low
TCGA-CN-6017	2.335	1	3.28504	3.85171	2.24702	3.93221	4.31072	8.23802	2.34822	0.74385	low
TCGA-CN-6018	1.5875	1	2.93171	3.79561	2.57684	3.65561	4.63265	6.08031	0.68119	1.18323	high
TCGA-CN-6020	0.56083	1	2.95472	4.02883	2.32708	3.15792	4.00489	7.06252	0.48529	1.41953	high
TCGA-CN-6021	0.75583	1	3.98974	4.45444	1.68808	4.47205	4.26662	7.05118	0.70277	0.23267	low
TCGA-CN-6023	4.33667	0	3.41191	3.38787	2.99518	4.08332	4.47914	8.76994	1.69159	2.35547	high
TCGA-CN-6988	0.87083	0	3.33966	3.94542	1.75699	4.00811	3.92669	7.13416	0.47942	0.58083	low
TCGA-CN-6989	2.6825	1	3.44923	3.67045	2.28216	3.77989	3.94261	7.75533	0.83332	1.04776	high
TCGA-CN-6992	2.91833	0	3.46625	3.81269	2.17037	4.31159	5.14456	8.01774	0.80283	1.50965	high
TCGA-CN-6994	3.23833	0	3.08822	4.49347	1.91166	2.79283	3.96423	7.87976	0.66781	1.24371	high
TCGA-CN-6995	0.30667	1	2.67178	4.46013	2.57236	3.67661	5.38115	8.50602	1.00176	4.07556	high
TCGA-CN-6996	1.45083	1	3.28385	3.68836	2.16637	3.90301	4.4958	7.11416	1.30951	0.79901	low
TCGA-CN-6997	2.705	1	3.19103	3.96683	2.35452	4.15415	4.75561	8.0959	0.49615	2.00963	high
TCGA-CN-A498	2.11583	1	3.02919	3.78644	2.34247	3.37451	3.73797	7.09208	0.68689	1.14269	high
TCGA-CN-A499	1.9625	0	3.49045	3.78228	2.28268	3.88637	5.14259	7.99697	1.85919	1.14456	high
TCGA-CN-A49B	2.475	0	3.2683	3.87469	2.49948	4.02099	5.02093	8.0768	1.36271	1.71011	high
TCGA-CN-A6V3	2.03167	0	3.4299	3.98691	2.64601	3.64222	4.49626	8.00893	0.9464	1.65815	high
TCGA-CN-A6V6	1.73833	0	3.57606	4.12409	2.38714	3.9745	4.60104	8.07174	2.4553	0.56875	low
TCGA-CQ-5323	4.01333	0	4.25593	4.40128	2.36493	4.45308	4.502	7.87028	1.17638	0.39234	low
TCGA-CQ-5325	1.79	1	2.93751	4.19604	2.18875	3.72424	4.64722	8.04263	1.65995	1.24578	high
TCGA-CQ-5326	0.24333	1	2.3896	3.48117	2.36716	3.64276	4.79409	7.25304	0.12984	4.46091	high
TCGA-CQ-5330	5.19333	0	3.24494	3.41315	1.99214	3.90542	4.47548	8.14302	1.36952	1.34282	high
TCGA-CQ-5331	3.83	0	3.1265	4.90532	1.89561	3.48734	4.31999	7.89035	1.30899	0.66593	low
TCGA-CQ-5332	0.8675	1	3.44802	4.13184	2.04771	4.14193	3.98525	8.0722	0.40532	0.92508	low
TCGA-CQ-6223	3.90917	0	2.46677	4.79046	2.39468	2.92386	4.57957	6.85766	0.53757	1.84693	high
TCGA-CQ-6229	3.2275	0	3.14584	4.34475	1.57035	3.61291	4.40856	8.05247	0.76756	0.9861	low
TCGA-CQ-7065	4.45667	0	3.17831	4.39699	1.38926	4.08069	5.51021	7.99017	0.02271	1.64014	high
TCGA-CQ-7069	3.4875	0	2.69608	4.21412	1.57535	3.56792	4.4491	7.23024	0.32229	1.24941	high
TCGA-CQ-7071	3.58917	0	2.72781	4.32849	2.12038	2.76118	4.36849	7.49366	0.57642	2.09632	high
TCGA-CQ-A4C6	3.70417	0	3.2211	3.76891	1.9317	3.22536	5.55586	8.10041	1.11379	2.65911	high
TCGA-CQ-A4CB	2.445	0	3.33491	3.76183	2.57398	3.80283	5.20822	7.35493	0.80901	1.94872	high

TCGA-CQ-A4CD	2.7975	0	4.00246	3.82406	2.7627	4.44451	4.74882	7.92862	0.655	1.16118	high
TCGA-CQ-A4CG	1.1775	1	2.98353	3.52066	2.05268	3.41481	5.35253	7.78531	0.2162	4.20095	high
TCGA-CQ-A4CI	2.60083	0	3.91545	3.71468	1.91638	4.15526	4.55412	7.01671	0.22656	0.63697	low
TCGA-CR-5243	7.01417	0	3.86507	4.17816	2.22079	4.22903	4.27806	8.0083	1.60225	0.45772	low
TCGA-CR-6473	3.08	1	3.73481	3.26454	3.00432	3.70817	4.48026	8.21478	0.80077	2.65858	high
TCGA-CR-6474	1.54417	0	2.96235	3.79599	1.83224	3.9419	3.79778	8.13216	0.53974	1.30175	high
TCGA-CR-6477	1.4075	1	2.45839	4.72864	1.97363	3.52002	3.89346	7.78827	0.61167	1.30403	high
TCGA-CR-6480	0.99083	0	3.45148	4.35673	2.15423	3.69114	4.1653	8.05898	0.89011	0.90403	low
TCGA-CR-6481	0.85167	0	3.0715	4.33746	2.30708	4.40539	3.69864	8.27686	2.50365	0.41977	low
TCGA-CR-6482	0.94417	0	3.11972	4.28109	2.59607	3.96899	4.33768	8.07602	2.11413	0.85876	low
TCGA-CR-6484	0.96917	0	2.74978	3.60809	2.71228	3.96877	3.84976	7.51856	1.44153	1.37492	high
TCGA-CR-6488	1.0375	0	3.43494	4.13319	2.07347	3.598	3.90265	7.53189	1.16006	0.6079	low
TCGA-CR-6491	1.8975	0	3.27222	4.37737	1.76653	3.11601	4.12416	7.58357	0.47965	0.95512	low
TCGA-CR-6492	1.31167	1	3.12713	4.47293	2.373	3.61336	4.44136	7.42988	0.82074	1.09813	high
TCGA-CR-7364	3.92833	0	2.34249	4.64855	2.31799	3.94548	4.45752	5.71694	0.6009	0.73919	low
TCGA-CR-7365	3.26083	0	3.11419	4.11884	1.45709	3.7923	4.19485	7.42941	0.18072	0.86349	low
TCGA-CR-7367	3.9425	0	3.32569	4.78925	2.03611	3.76423	4.11142	7.36675	0.97971	0.48769	low
TCGA-CR-7372	2.0775	0	3.28776	4.9322	1.75487	3.62268	3.44528	8.36031	0.40661	0.64391	low
TCGA-CR-7373	2.43333	0	2.97638	3.84215	2.38011	4.13266	4.8325	7.80814	1.29469	1.58864	high
TCGA-CR-7379	2.83583	1	3.37411	4.4828	1.61884	4.35547	4.28592	8.27778	0.17942	0.83747	low
TCGA-CR-7380	1.65917	0	3.04572	4.32158	2.16145	3.90387	3.54447	7.63085	0.47289	0.83757	low
TCGA-CR-7382	2.17917	1	3.10755	3.81092	2.2865	3.70226	4.97256	8.02534	1.44232	1.81578	high
TCGA-CR-7383	1.42667	0	3.33442	3.86662	2.49458	3.5905	4.25356	8.40604	0.97734	1.87113	high
TCGA-CR-7388	2.25333	0	3.15336	4.48945	2.43053	3.36538	4.10966	7.62806	1.4096	0.86836	low
TCGA-CR-7391	2.49917	0	2.84919	5.10253	1.69251	3.4796	3.45467	7.71769	1.38249	0.3878	low
TCGA-CR-7393	2.71833	0	3.04545	4.7365	1.93557	3.46456	3.51027	7.67402	1.87597	0.37206	low
TCGA-CR-7395	2.54583	0	2.94793	4.83958	2.00229	3.89065	4.16316	7.1933	1.41633	0.44949	low
TCGA-CR-7399	0.49583	0	3.1262	4.35402	2.45491	5.6809	4.26645	8.10581	1.39198	0.54215	low
TCGA-CR-7402	2.49417	0	3.31379	4.70904	2.72855	4.91437	4.28164	7.20461	0.71857	0.55798	low
TCGA-CV-5431	1.42917	0	3.37081	3.87878	2.07944	4.21268	4.53074	7.36568	1.96037	0.49055	low
TCGA-CV-5432	10.7592	1	3.69696	4.09281	1.21643	4.32677	4.0831	8.83896	0.51062	0.6545	low
TCGA-CV-5441	7.90083	0	3.25157	4.19049	1.72831	5.66166	4.37155	4.73783	3.01308	0.03437	low
TCGA-CV-5442	6.37083	0	3.78999	4.0937	1.90267	4.33535	3.98742	7.38832	0.26359	0.48267	low
TCGA-CV-5973	7.23	0	3.003	4.4365	1.29364	3.01272	4.21893	7.20469	0.22128	0.87504	low
TCGA-CV-5976	4.04583	0	3.6689	3.8521	1.97424	3.17898	4.5196	7.49016	0.2463	1.31047	high
TCGA-CV-5977	5.0375	1	3.59194	3.69406	1.85717	3.76702	4.95268	8.25645	0.27773	1.95895	high
TCGA-CV-6433	1.755	0	3.12916	4.17824	2.64358	4.4722	5.62777	8.06779	1.87196	1.56834	high
TCGA-CV-6436	5.19917	1	2.77927	4.23079	1.24997	2.81832	4.26062	7.49499	0.83691	1.05208	high
TCGA-CV-6933	7.50417	1	2.86708	4.47762	2.03693	4.30909	3.73955	7.37209	0.59037	0.64558	low
TCGA-CV-6935	0.8075	1	3.11747	3.74347	2.52961	2.24959	4.42827	7.76765	0.14888	4.64065	high
TCGA-CV-6937	1.70833	1	2.61994	4.57231	2.37856	2.90893	4.66717	8.77443	0.90464	4.0678	high
TCGA-CV-6939	1.82333	1	3.19365	4.09102	2.76705	3.92938	4.43403	7.8669	1.32589	1.36601	high
TCGA-CV-6940	2.20083	1	3.13068	3.87317	1.47457	3.68661	4.34875	8.29786	0.53986	1.4012	high
TCGA-CV-6942	11.7225	1	3.26805	4.15403	2.33494	4.25195	4.01117	7.58743	1.83265	0.48723	low
TCGA-CV-6945	1.00167	1	2.38593	4.50331	3.15426	3.40612	4.01586	7.70637	0.08485	4.13733	high
TCGA-CV-6951	2.505	1	3.52792	4.0912	1.41052	3.72829	4.22726	7.37841	0.21517	0.63129	low
TCGA-CV-6955	0.91417	1	2.48409	5.24487	1.85357	2.57644	3.92328	6.41696	0.82067	0.63227	low
TCGA-CV-6956	0.59417	1	3.20827	3.86557	1.9201	4.64618	4.17585	7.24504	0.23722	0.76059	low
TCGA-CV-6960	2.36	1	3.63471	4.43847	1.46453	4.33037	4.37689	8.10039	0.89864	0.44898	low
TCGA-CV-6961	0.20833	1	2.66021	4.20765	2.97299	3.09013	4.70658	7.28966	0.27902	4.12945	high
TCGA-CV-7089	5.39833	0	2.9186	4.9152	1.93067	3.34163	4.44191	7.62567	0.53374	1.11089	high
TCGA-CV-7091	9.25583	1	3.24003	4.18127	1.97785	3.89903	3.8584	6.50292	1.29521	0.32227	low
TCGA-CV-7097	1.05417	1	3.20353	4.12304	2.35693	3.92901	4.52165	8.0175	0.62558	1.63404	high
TCGA-CV-7101	0.43833	1	2.89632	3.92047	1.8306	3.80325	3.64463	7.78394	0.63297	1.01854	low
TCGA-CV-7102	0.15333	1	2.60521	3.35844	2.45103	3.57595	3.89181	7.24326	0.47779	2.40254	high
TCGA-CV-7178	5.93	1	3.23839	3.68659	2.38003	3.44782	4.10786	7.80182	1.06219	1.44744	high
TCGA-CV-7183	10.8983	0	2.54544	5.08931	1.98088	3.19152	3.58238	6.30394	0.04423	0.64087	low
TCGA-CV-7235	6.425	1	3.88389	3.63924	2.56444	3.8048	3.84086	7.92549	0.84883	0.93143	low
TCGA-CV-7236	0.39417	0	3.0442	3.92261	2.31274	3.57657	4.48713	7.65174	0.32299	2.13676	high
TCGA-CV-7238	7.46583	0	3.28186	4.09857	2.21146	3.78334	4.65955	8.13712	1.95845	0.92476	low
TCGA-CV-7247	1.58	1	3.46216	3.43975	2.44324	3.76084	4.56016	7.35565	0.0883	2.00831	high
TCGA-CV-7254	3.99417	1	2.79158	4.09948	2.35062	3.82879	4.50896	8.34968	1.26358	1.99919	high
TCGA-CV-7255	0.175	0	3.28976	4.56205	2.05837	3.63298	4.42088	7.86052	0.24718	1.23082	high
TCGA-CV-7263	1.53333	1	4.02563	4.42048	2.0432	4.16925	4.14153	7.70251	0.67787	0.41534	low
TCGA-CV-7411	7.43833	1	2.49926	4.82915	1.82669	2.93422	3.56976	7.09817	0.31509	0.97557	low
TCGA-CV-7413	0.805	1	2.48275	4.4174	2.49778	3.25399	4.72104	7.84415	0.66436	3.32691	high
TCGA-CV-7414	0.03833	1	3.38905	4.30968	1.65072	3.31696	4.28153	8.88287	0.66762	1.45434	high
TCGA-CV-7415	1.9025	1	3.11089	4.08684	2.32542	4.04679	4.0236	7.741	0.48485	1.22908	high
TCGA-CV-7421	0.00583	1	2.71634	4.24343	1.76143	4.29554	3.62564	7.72864	0.16145	0.94843	low
TCGA-CV-7423	8.37417	1	2.98461	4.87588	2.55268	2.89664	3.92279	7.19151	0.08503	1.43172	high
TCGA-CV-7427	13.0308	1	2.76334	4.38841	2.11582	3.52527	4.34257	7.67237	1.23839	1.1706	high
TCGA-CV-7428	4.57417	1	4.02167	4.14119	2.2962	3.82137	4.99168	7.37981	1.10345	0.66433	low
TCGA-CV-7430	1.355	1	2.44732	4.03127	2.42827	3.30493	3.6553	6.99211	0.05743	2.04665	high
TCGA-CV-7432	7.03583	1	3.62689	3.86019	1.8687	3.59332	4.69332	7.54725	0.07693	1.30523	high
TCGA-CV-7433	1.645	1	2.8113	4.27933	2.4515	3.85051	4.22772	7.86867	1.33686	1.27246	high

TCGA-CV-7434	0.59667	1	3.38923	3.3359	2.12356	3.45754	4.51348	8.05398	0.2966	2.58307	high
TCGA-CV-7435	12.8125	1	3.03552	3.74622	1.86647	3.74711	4.35279	6.44625	0.58137	0.78604	low
TCGA-CV-7438	0.53083	1	3.42698	3.45185	2.01745	3.88579	4.42047	5.62901	0.4545	0.52193	low
TCGA-CV-7440	1.8475	1	2.6918	3.65744	2.42388	3.85271	4.53906	7.20705	0.6542	2.13198	high
TCGA-CV-A45P	1.74917	1	2.99302	4.61764	1.89648	3.09	4.06387	6.30352	0.53629	0.57736	low
TCGA-CV-A45Q	14.1042	0	3.47106	4.45408	0.59615	4.96927	4.2436	7.43011	0.40622	0.20233	low
TCGA-CV-A45R	15.0025	1	3.24416	4.10419	2.03634	4.3858	4.04213	7.35837	0.83868	0.5848	low
TCGA-CV-A45T	13.2942	1	3.05978	5.01219	1.97849	3.76799	4.90525	7.82704	2.77471	0.40592	low
TCGA-CV-A45U	2.95417	1	3.33306	4.45985	2.91628	3.74608	4.87142	7.72702	1.50248	1.28194	high
TCGA-CV-A45V	0.0875	1	2.97794	4.13473	2.06157	3.30979	4.31901	7.71918	1.02158	1.33409	high
TCGA-CV-A45Y	7.4	1	3.61418	4.3249	2.34076	4.0244	3.98121	7.4791	2.01943	0.32797	low
TCGA-CV-A460	5.03167	1	3.57555	4.03465	2.79241	4.87981	5.65251	7.65495	1.94952	0.90851	low
TCGA-CV-A461	5.65083	1	3.47599	4.34996	2.34843	5.40849	4.79783	8.22741	2.26057	0.40173	low
TCGA-CV-A463	0.06333	0	3.01161	4.1445	2.40875	3.64354	4.02296	7.04729	0.6088	1.06088	high
TCGA-CV-A465	0.58833	1	2.96254	4.77634	1.82922	3.06825	5.14498	7.3048	0.24822	1.64698	high
TCGA-CV-A468	1.27	1	3.32719	4.31551	1.974	4.25861	4.60909	7.46453	2.5097	0.3195	low
TCGA-CV-A6JE	2.94333	1	3.27939	4.18839	1.36832	4.26495	4.59712	7.91661	1.87599	0.40984	low
TCGA-CV-A6JM	0.53083	0	3.34053	5.03385	0.86264	4.55057	4.07165	7.91821	0.46473	0.26119	low
TCGA-CV-A6JN	2.48	1	2.81544	4.53054	1.75762	3.88814	4.87012	8.51127	0.9213	1.66367	high
TCGA-CV-A6JO	0.53917	0	3.12974	4.27767	1.80501	3.52677	4.30895	7.8006	1.20407	0.84134	low
TCGA-CV-A6JT	2.3325	0	3.1993	4.31134	1.50881	3.91705	4.23948	7.26584	0.99151	0.47071	low
TCGA-CV-A6JU	0.30083	0	3.77742	4.53863	1.41925	5.52711	4.47157	6.91589	0.55289	0.16483	low
TCGA-CV-A6JY	1.76833	0	3.19843	5.0476	2.19012	3.40173	4.52278	7.67013	0.38271	1.10195	high
TCGA-CV-A6K0	1.65917	0	3.22101	4.12077	2.0981	3.80245	4.37696	7.66309	1.94695	0.62069	low
TCGA-CV-A6K1	1.875	1	3.37955	3.53318	1.99202	4.13239	5.46481	8.54235	0.66815	2.83953	high
TCGA-CX-7082	0.03	0	3.64052	4.43101	1.88575	4.22945	4.60017	7.82861	0.50388	0.698	low
TCGA-CX-7085	0.87917	0	2.80215	4.46271	2.07268	3.46073	3.68871	7.5107	1.82231	0.5665	low
TCGA-CX-7086	1.56833	0	3.76232	4.59725	1.71771	3.88293	3.76812	7.28415	0.48747	0.31795	low
TCGA-CX-A4AQ	4.25667	1	3.91539	4.49953	2.27774	5.03415	5.26035	8.95347	2.8139	0.41268	low
TCGA-D6-6515	1.10333	0	2.62622	3.8432	2.19785	4.51858	4.40862	8.23201	2.43275	0.93795	low
TCGA-D6-6825	1.34417	1	2.40309	4.17847	2.22401	3.37669	4.51368	7.77764	1.67618	1.76483	high
TCGA-D6-6827	1.555	0	3.18359	3.98463	2.20265	4.38021	4.36606	8.29079	0.49528	1.53563	high
TCGA-D6-A4Z9	1.47583	0	3.27403	3.97519	2.46049	2.76831	4.12831	7.87669	0.88543	1.89417	high
TCGA-D6-A6EO	2.0775	0	3.5371	3.8491	1.28897	4.94293	5.12548	7.98404	1.00175	0.58971	low
TCGA-D6-A6EP	1.16083	0	4.11339	5.23048	1.60225	3.92465	4.42311	9.55046	0.48851	0.68328	low
TCGA-D6-A6EQ	1.0075	0	2.93889	4.19746	1.60862	3.63158	4.67923	8.26026	1.12514	1.33019	high
TCGA-D6-A74Q	1.94333	0	3.4195	3.65871	1.695	5.03448	4.16447	7.96219	2.71675	0.24272	low
TCGA-DQ-5629	2.57583	0	3.25906	3.53745	2.06089	3.89829	3.73998	7.81812	0.12369	1.40208	high
TCGA-DQ-7588	1.16917	0	3.00827	4.7521	2.36814	4.05722	5.00024	7.203	0.07401	1.45612	high
TCGA-DQ-7592	3.12917	0	3.04506	4.03853	1.82449	4.21789	4.09801	7.4151	0.59743	0.7759	low
TCGA-F7-A50G	1.68667	0	2.9755	4.63681	2.07509	3.38886	4.07939	8.09268	1.32119	0.95293	low
TCGA-F7-A50I	0.25167	0	2.81835	3.20517	2.5545	3.65788	4.22648	8.3021	0.99083	3.52194	high
TCGA-F7-A50J	2.5925	0	2.94946	3.95062	2.22626	2.93363	5.20315	7.47017	1.01367	2.56585	high
TCGA-F7-A61V	2.0775	0	3.73767	4.19729	1.61001	3.97043	4.77228	7.76404	0.77757	0.63497	low
TCGA-F7-A622	0.9825	0	3.1975	3.74386	1.34593	3.70962	5.43416	8.01584	0.14701	2.29787	high
TCGA-H7-7774	1.11417	0	2.47244	4.84716	1.80396	3.07591	4.03603	6.76427	0.61749	0.8513	low
TCGA-H7-8501	1.26167	0	2.86038	3.29447	2.40565	3.82278	5.39408	8.52159	2.22619	3.08045	high
TCGA-H7-A76A	1.74417	0	3.52913	4.38663	2.09834	4.21857	4.63293	8.18673	3.42329	0.27321	low
TCGA-HD-7753	2.37083	0	2.94339	3.41526	2.0156	3.68877	3.73302	7.13015	0.66112	1.09747	high
TCGA-HD-7754	2.14333	0	3.22915	4.03008	1.79039	3.7611	4.20856	7.43884	0.38171	0.93848	low
TCGA-HD-7831	1.82583	0	2.82423	3.67357	2.00058	2.70994	3.35812	7.34995	0.33286	1.64737	high
TCGA-HD-8224	1.22083	1	3.61244	4.10015	2.25896	3.1439	4.28338	7.54594	0.4823	1.1908	high
TCGA-HD-8634	1.05417	1	3.26276	4.6581	1.99977	3.01103	4.1401	6.51244	0.61999	0.56273	low
TCGA-HD-A4C1	0.03	0	3.04228	4.16469	2.3827	3.36149	4.30203	7.22289	0.55995	1.43406	high
TCGA-HD-A634	0.35583	1	3.13632	3.89471	2.84005	4.06321	4.88083	9.15102	0.08305	6.36781	high
TCGA-HD-A6HZ	0.30417	0	2.70321	3.92333	2.3659	4.17611	4.18824	7.6684	1.48798	1.1403	high
TCGA-HL-7533	2.89333	0	4.37589	2.77259	2.68716	3.88304	4.6581	7.81427	1.24838	1.20316	high
TCGA-IQ-7632	1.2075	0	2.64891	4.73053	2.24058	3.93083	4.1263	8.21318	0.13854	1.93786	high
TCGA-IQ-A61I	0.00583	1	3.45235	4.44431	2.9193	4.75889	5.03031	8.72928	2.71654	0.80341	low
TCGA-IQ-A6SG	1.585	0	3.2568	4.34267	2.37108	3.57815	5.20539	8.17879	0.26667	2.88474	high
TCGA-KU-A6H7	1.60417	0	3.29859	3.36293	2.34916	4.05008	5.45787	8.30796	2.51259	1.56809	high
TCGA-KU-A6H8	0.895	1	3.31121	4.09957	2.32996	3.73903	4.13452	8.0945	1.57773	0.89739	low
TCGA-MT-A51X	0.6625	0	2.99746	5.0681	1.99292	3.60649	4.253	7.55378	0.58327	0.78767	low
TCGA-MT-A67A	2.5025	0	2.91659	3.75117	1.7329	2.94804	4.60081	4.35247	1.01074	0.35933	low
TCGA-MT-A67F	1.05083	0	2.99451	4.41014	2.20631	3.99132	4.56763	7.72874	2.90675	0.45571	low
TCGA-MT-A7BN	1.28417	0	3.41636	5.23721	2.2153	5.09265	3.98917	8.21936	1.29409	0.30251	low
TCGA-P3-A5Q6	1.31417	1	2.70038	3.56905	1.42976	3.66532	4.57081	7.38721	0.24769	1.78352	high
TCGA-P3-A5QA	5.97333	0	3.52861	3.88804	2.30766	3.27281	4.76663	7.43982	0.35618	1.74139	high
TCGA-P3-A5QE	4.26833	0	3.5643	4.05676	2.67896	4.60972	4.54694	8.10948	3.08877	0.40732	low
TCGA-P3-A5QF	0.90333	1	3.1157	3.61124	1.91321	4.21599	5.04123	8.15102	0.57898	2.15711	high
TCGA-P3-A6T0	1.5825	0	3.00592	3.9383	2.30748	2.92834	4.39661	6.54119	0.45416	1.46615	high
TCGA-P3-A6T3	1.58	1	3.10862	4.14303	1.82712	3.10412	4.4939	7.51745	0.09712	1.72899	high
TCGA-P3-A6T5	2.415	1	3.48027	4.13069	1.63946	3.24475	4.34242	8.44191	0.51725	1.35255	high
TCGA-P3-A6T7	1.33333	1	2.42659	4.17796	2.01616	3.09911	4.73999	6.87733	0.72093	1.92111	high
TCGA-QK-A64Z	1.755	1	3.21748	3.37232	2.5666	3.13711	4.68308	7.17377	0.45646	2.7626	high

TCGA-QK-A652	1.76583	0	3.20699	3.8693	1.65492	3.77722	4.86128	6.87117	1.7536	0.51936	low
TCGA-QK-A6IF	1.9275	0	3.61532	3.88098	2.52385	5.23659	4.49315	7.31389	3.18191	0.1953	low
TCGA-QK-A6IG	0.6075	1	3.51178	3.62644	1.87298	4.25553	5.78209	8.70407	1.46754	1.90585	high
TCGA-QK-A6IH	1.7875	0	3.51789	4.23527	1.48824	3.81897	4.38264	8.25635	0.27894	0.95806	low
TCGA-QK-A6II	0.7775	1	2.79943	5.34805	1.56257	3.35686	4.29683	7.60274	0.60393	0.70403	low
TCGA-QK-A6V9	2.28083	0	3.73294	4.03889	2.74217	3.98155	4.85595	8.55896	2.18709	1.05034	high
TCGA-QK-A6VB	1.755	0	3.6996	4.55917	1.74446	4.04548	5.09471	8.66614	1.21548	0.84727	low
TCGA-QK-A6VC	1.6425	0	3.03984	4.36974	2.25049	3.86469	4.45825	7.76136	2.00004	0.71166	low
TCGA-QK-A8Z7	1.07333	0	4.62086	4.18446	2.18545	4.89057	4.28897	7.38305	1.26496	0.17481	low
TCGA-QK-AA3J	1.27583	0	3.48968	4.34266	1.99097	3.42452	3.65776	7.26576	0.97353	0.45877	low
TCGA-RS-A6TP	1.4125	0	3.64145	4.73093	2.68309	4.35556	4.40442	7.28035	0.74465	0.5709	low
TCGA-T2-A6WX	0.5725	1	3.17224	3.98665	2.2388	4.07466	6.16499	8.762	1.81475	2.84861	high
TCGA-T2-A6WZ	1.325	1	3.87242	3.98006	1.20772	4.55857	4.23094	8.07544	0.82178	0.35595	low
TCGA-T2-A6X0	0.59167	0	3.354	3.9268	2.4127	4.95359	4.6914	8.0062	1.42819	0.83614	low
TCGA-T3-A92N	0.26	1	3.0829	3.77812	2.53501	3.09885	4.46868	7.52358	1.04878	2.02369	high
TCGA-TN-A7HI	1.1275	0	3.7586	4.53476	2.02146	4.73277	4.41646	8.24497	2.14136	0.28495	low
TCGA-UF-A718	5.39583	0	3.74951	3.92501	2.02279	4.14069	3.85719	7.2425	1.25615	0.34397	low
TCGA-UF-A719	4.5525	0	2.99983	4.31731	2.05082	4.46103	4.20054	8.758	0.63712	1.4371	high
TCGA-UF-A7JA	6.20083	0	3.25593	4.58451	2.96558	3.44347	3.90352	7.06312	1.27636	0.74845	low
TCGA-UF-A7JC	1.495	1	2.9063	4.64951	1.64169	2.95133	4.17712	7.43377	0.18825	1.17961	high
TCGA-UF-A7JF	4.61583	0	2.96588	4.68978	1.82562	3.78157	4.91675	8.58614	0.79218	1.68538	high
TCGA-UF-A7JH	2.4525	0	3.49244	4.27172	2.2823	4.15194	3.79358	9.11696	1.26852	0.96819	low
TCGA-UF-A7JJ	1.50333	0	2.85192	4.32691	2.18405	3.61962	4.71076	7.92332	2.10354	1.02222	low
TCGA-UF-A7JO	1.7275	1	2.61787	3.1468	2.1876	3.63974	5.14676	8.55995	2.06485	3.63422	high
TCGA-UF-A7JS	1.86167	1	3.49663	4.43761	0.97823	5.38592	5.28148	8.05055	0.93289	0.36973	low
TCGA-UF-A7JV	0.24667	1	3.21236	3.95847	2.44017	3.67845	6.82082	8.07946	0.83652	5.6808	high
TCGA-UP-A6WW	1.41833	0	2.3704	4.11953	2.34523	4.63051	4.78386	8.32434	1.19354	2.29162	high

Supplementary Table S2. The riskscores of HNSCC patients in the TCGA HNSCC test cohort.

ID	futime	fustat	MAP2K7	MAPK3	MAPK9	ORAI1	PSMA1	UBB	ZAP70	Riskscore	Risk
TCGA-BA-4075	0.775	1	2.30486	3.87163	2.37415	3.77856	5.56881	9.24554	0.05766	14.6805	high
TCGA-BA-4077	3.10417	1	2.89015	4.56208	2.57004	3.93344	4.93502	8.21216	1.09452	2.01473	high
TCGA-BA-5151	1.97667	0	2.82765	4.40837	2.2275	3.80254	4.03473	7.73698	0.7988	1.16076	high
TCGA-BA-5153	4.82333	1	3.06516	4.1333	2.66248	4.22953	4.27699	8.23373	2.5982	0.75591	low
TCGA-BA-5555	1.42333	0	3.17868	4.48566	2.33953	4.66858	3.70821	5.22808	0.72341	0.17523	low
TCGA-BA-5557	1.70583	0	2.64545	4.78819	1.6756	3.28678	3.71492	7.12353	0.62317	0.67396	low
TCGA-BA-5559	5.7025	1	3.90748	4.0514	2.41612	4.51431	4.41138	7.87269	2.991	0.24822	low
TCGA-BA-6868	1.2925	1	1.84763	4.40024	2.46913	3.6879	5.43941	7.55074	0.23196	6.60805	high
TCGA-BA-6872	1.05083	1	3.22216	3.96012	1.74407	3.99467	3.75914	8.45588	0.50936	1.08057	high
TCGA-BA-7269	3.485	0	2.51143	3.90438	1.4916	3.52951	4.3756	8.70462	0.2614	3.24866	high
TCGA-BA-A4II	2.51333	0	3.54194	4.51204	2.4183	3.87523	4.43851	7.10069	0.96162	0.59179	low
TCGA-BA-A6D8	2.32667	0	3.76669	4.88898	1.36903	5.02106	5.89404	8.56012	0.52399	0.731	low
TCGA-BA-A6DB	0.59167	0	3.08651	4.24643	1.74277	3.83076	4.5403	7.80837	2.10379	0.55843	low
TCGA-BA-A6DD	0.47333	1	2.94784	4.52382	2.07634	3.79314	4.744	8.13777	1.45563	1.16082	high
TCGA-BA-A6DE	1.20417	0	3.55236	3.96106	2.1622	4.17289	5.19881	6.95134	0.41666	1.01577	low
TCGA-BA-A6DL	1.70583	0	2.91683	4.48242	1.97786	3.78301	4.75171	7.84235	1.76046	0.86707	low
TCGA-BB-4217	0.51167	0	3.22188	4.29164	2.27026	4.42803	4.40712	7.69351	3.15053	0.29251	low
TCGA-BB-4223	8.8175	0	3.90469	3.67196	2.87285	4.24766	4.43128	5.77261	2.56705	0.1921	low
TCGA-BB-4224	0.76083	0	3.21192	4.4603	2.27584	4.47277	5.14354	7.66186	0.40881	1.33949	high
TCGA-BB-4227	0.36667	0	3.20965	4.49504	2.89943	2.96739	3.75823	7.81295	0.17004	2.09121	high
TCGA-BB-8596	5.91583	0	3.08487	3.86114	1.93378	3.3662	4.25412	7.40733	1.39213	0.89913	low
TCGA-BB-8601	1.70833	0	3.58905	3.09983	1.76135	3.93005	5.14102	7.61129	0.15641	1.97057	high
TCGA-BB-A5HU	2.14083	0	3.32967	4.01128	1.80775	3.56503	4.29266	5.23029	0.5296	0.32022	low
TCGA-BB-A5HY	0.87917	1	3.1524	3.30006	2.97122	4.88988	5.59849	7.74167	0.4282	4.00791	high
TCGA-BB-A6UM	1.07583	0	3.44857	4.69398	2.25252	4.19209	5.1313	8.23394	1.56437	0.84865	low
TCGA-BB-A6UO	0.73333	1	2.99712	4.1018	2.03899	3.57232	4.2879	7.66279	0.31052	1.59231	high
TCGA-C9-A47Z	0.5225	1	3.1067	4.2424	1.83503	2.93727	4.40747	6.64088	0.43806	0.94702	low
TCGA-CN-4722	4.06	0	2.58905	4.93231	1.81135	3.16297	3.66162	6.76621	0.62176	0.60957	low
TCGA-CN-4723	4.65083	0	2.91749	3.95944	2.562	3.64653	4.80166	8.01415	1.37273	2.20051	high
TCGA-CN-4727	4.27083	0	3.16807	4.56365	1.69382	4.15925	4.49502	6.99625	0.8227	0.46387	low
TCGA-CN-4729	1.07333	0	2.4292	4.11416	2.34322	3.65612	3.84079	7.3821	0.64009	1.6686	high
TCGA-CN-4731	2.7325	1	2.36923	3.69334	1.74946	3.6988	4.65563	7.77038	0.74354	2.52438	high
TCGA-CN-4737	1.71083	0	3.31312	4.00933	1.78365	3.80111	4.04586	7.61709	0.83208	0.71611	low
TCGA-CN-4738	1.19333	1	2.90918	4.16502	2.34402	3.18839	4.20453	7.41155	1.32296	1.20965	high
TCGA-CN-4740	2.29667	1	2.62855	4.23598	2.38222	3.73489	3.86592	8.13681	0.74331	1.8789	high
TCGA-CN-4741	6.12917	0	3.29046	4.17373	2.66057	4.09098	4.21296	8.31432	0.81078	1.53512	high
TCGA-CN-5361	5.80417	1	2.56946	4.06084	2.45996	3.73497	3.56951	6.93905	0.59592	1.16308	high
TCGA-CN-5364	1.35	1	2.89815	3.90894	1.92212	3.03338	4.76883	7.50641	0.56698	2.23124	high
TCGA-CN-5365	0.96083	1	1.88052	4.26037	2.19794	2.5057	4.23484	8.05117	0.572	5.67252	high
TCGA-CN-5366	0.98583	1	2.80579	3.71068	1.72083	3.15726	3.97478	8.4047	0.74338	2.16981	high
TCGA-CN-5367	0.96333	1	2.82423	3.84521	2.54724	4.56552	4.62254	7.79137	0.25157	2.40959	high
TCGA-CN-5369	1.04	1	2.65155	4.44232	2.49181	3.59844	4.46555	7.33638	1.73384	1.07111	high
TCGA-CN-5373	4.33667	0	2.77132	4.49593	1.7929	2.79633	3.96924	7.71861	1.05525	1.12732	high
TCGA-CN-5374	4.74167	1	3.48887	4.29661	2.13572	3.95541	3.69924	7.72295	2.80092	0.22693	low
TCGA-CN-6013	1.99	1	3.21873	4.71261	2.45769	3.33611	3.91218	7.55498	2.35641	0.43221	low
TCGA-CN-6016	3.95	0	2.62028	4.1397	2.05255	3.41658	3.53189	7.26412	1.02565	0.90542	low
TCGA-CN-6019	2.84167	0	3.36864	4.27866	1.98848	3.57896	3.923	7.85807	1.1727	0.66978	low
TCGA-CN-6022	0.76917	1	3.0403	4.70867	2.06651	3.67665	4.19039	7.8303	0.68015	0.9794	low
TCGA-CN-6024	0.9225	1	3.70335	3.93106	1.90712	3.73729	4.49547	9.57574	0.12013	2.74789	high
TCGA-CN-6998	0.9775	1	3.43681	4.62083	1.85678	2.66582	3.85731	7.42412	0.26843	0.84835	low
TCGA-CN-A497	2.91583	0	3.46777	3.77493	2.6478	4.66193	4.59122	7.64099	1.70485	0.74636	low
TCGA-CN-A49A	1.44	1	3.44737	4.71763	2.24612	3.46885	4.32578	7.609	0.37009	0.96681	low
TCGA-CN-A49C	1.76583	0	3.58745	4.39988	2.29671	4.50736	3.83334	8.05845	0.90646	0.54058	low
TCGA-CN-A63T	0.61583	0	2.80026	3.75746	2.02798	4.32585	5.3452	6.03949	1.60063	0.67007	low
TCGA-CN-A63U	2.63917	0	3.3102	3.10502	2.18669	3.91632	4.37125	8.49013	3.60662	0.65997	low
TCGA-CN-A63V	1.85917	0	3.42642	3.78858	2.05243	3.61476	3.64143	7.64895	0.70128	0.82016	low
TCGA-CN-A63W	1.0325	1	3.46752	3.65065	2.04791	4.00846	5.01407	7.84674	2.35112	0.71869	low
TCGA-CN-A641	1.005	0	2.84482	4.1246	2.35184	4.26552	4.83477	6.46677	1.63143	0.63754	low
TCGA-CN-A642	0.22417	1	3.70641	3.6245	2.64612	3.87901	4.92091	8.26552	1.20679	1.81614	high
TCGA-CN-A6UY	1.95167	0	3.34696	4.19427	2.54942	4.6722	5.083	9.06343	3.43443	0.7044	low
TCGA-CN-A6V7	1.62583	0	4.30971	3.66082	2.4048	4.43885	4.37345	8.38292	0.92431	0.75296	low
TCGA-CQ-5324	4.36083	0	2.88336	4.39883	2.19761	2.64897	4.23726	7.34437	0.54215	1.75535	high
TCGA-CQ-5327	4.54417	0	3.23941	4.09488	2.30726	4.04692	4.25423	7.892	0.88896	1.09883	high
TCGA-CQ-5329	5.86667	0	3.04074	4.43321	1.83512	4.54729	4.39154	8.41995	0.73178	1.00231	low
TCGA-CQ-5333	0.93333	1	3.26932	4.38025	2.4162	4.02509	5.13913	8.2315	1.61952	1.28794	high
TCGA-CQ-5334	0.35333	1	2.95789	4.67828	2.07096	4.3863	4.95806	7.99029	1.56832	0.8318	low
TCGA-CQ-6218	3.43	0	2.84721	4.18603	1.895	3.26227	4.54802	7.95907	0.39323	2.21749	high
TCGA-CQ-6219	1.31167	1	3.11233	4.2738	2.47624	3.54357	5.12886	8.45467	1.02446	2.76283	high
TCGA-CQ-6220	2.69667	1	2.78278	4.9851	1.97837	3.39047	4.28181	7.20941	0.60417	0.8764	low
TCGA-CQ-6221	2.7375	0	2.93112	3.41182	2.40346	3.46857	3.41823	8.35656	0.2041	2.91073	high
TCGA-CQ-6222	5.51917	0	2.89977	4.35606	2.21254	3.92722	4.17505	7.44221	1.35032	0.76652	low
TCGA-CQ-6224	4.71167	0	2.75287	3.20527	2.2296	3.69361	4.29869	7.72737	0.58018	2.86063	high

TCGA-CQ-6225	1.10333	1	2.94739	4.18756	2.14364	4.04191	4.47995	3.43013	0.2368	0.20593	low
TCGA-CQ-6227	0.35333	1	3.32989	4.43972	1.98055	4.52361	4.3346	7.63434	0.4337	0.68064	low
TCGA-CQ-6228	1.24833	1	3.46947	4.1321	2.35039	3.19539	4.67729	7.60094	0.24996	1.85582	high
TCGA-CQ-7063	5.83917	0	3.13248	4.23353	1.9089	3.41896	4.46524	7.66003	1.57333	0.80507	low
TCGA-CQ-7068	3.58333	0	3.45537	3.89998	2.10066	4.02244	4.64618	7.76657	1.98777	0.62981	low
TCGA-CQ-7072	6.45833	0	3.06434	3.47332	2.2111	3.25688	4.22226	7.55255	1.79164	1.18359	high
TCGA-CQ-A4C7	0.96667	1	3.62776	3.25963	1.85616	3.89395	4.66868	7.51441	0.70538	1.1215	high
TCGA-CQ-A4C9	1.93583	0	3.49703	4.56805	2.22577	4.15859	4.314	7.3668	0.43838	0.65247	low
TCGA-CQ-A4CE	2.45583	1	3.25837	4.56263	1.76052	3.32993	4.6795	3.85999	1.21925	0.12363	low
TCGA-CQ-A4CH	1.0375	0	3.70481	4.46328	2.01573	4.23381	4.3676	7.19872	0.72062	0.42148	low
TCGA-CR-5247	0.98	0	2.82666	4.32816	1.77427	4.06354	4.34325	7.70509	0.12333	1.31139	high
TCGA-CR-5248	4.5525	0	3.74216	4.82305	2.46293	4.38359	3.62925	7.8468	3.39864	0.11702	low
TCGA-CR-5249	3.15333	0	3.26929	4.19847	2.43724	4.07568	3.97232	7.67126	2.05867	0.49776	low
TCGA-CR-5250	2.1875	0	3.87029	4.20184	2.26613	4.54165	4.10308	8.19894	2.48599	0.27481	low
TCGA-CR-6467	4.865	0	4.27344	3.96446	3.03068	4.39817	4.58603	8.03906	2.57515	0.42437	low
TCGA-CR-6470	4.16417	1	3.36958	3.49246	2.30962	4.11513	4.44106	8.50493	2.17122	1.05857	high
TCGA-CR-6471	3.29083	0	3.08343	4.61172	2.64787	3.78895	3.85474	7.55008	1.36967	0.72177	low
TCGA-CR-6472	2.87417	0	3.2898	4.01227	2.451	3.85108	3.74831	7.9195	0.46223	1.2541	high
TCGA-CR-6478	0.50083	0	2.79854	4.39766	2.86638	3.91019	4.03694	8.28505	1.26316	1.7434	high
TCGA-CR-6487	0.64083	0	3.80393	3.61781	2.60191	4.42903	4.57717	8.65317	3.21442	0.54036	low
TCGA-CR-6493	0.77167	0	3.55709	4.27639	2.17826	3.50092	4.61744	6.7456	0.17416	0.87063	low
TCGA-CR-7368	3.40833	1	3.42838	3.7111	1.9275	3.57465	4.40567	7.34042	1.27644	0.76234	low
TCGA-CR-7369	2.98417	0	3.42833	4.01169	1.83376	3.80956	4.59266	7.29608	0.68783	0.80269	low
TCGA-CR-7370	0.2875	1	3.44714	4.19929	2.19523	3.60637	4.71444	7.87647	0.67602	1.36042	high
TCGA-CR-7371	0.2575	0	2.93213	4.36166	2.45007	4.24527	4.49657	7.67233	1.32397	1.00151	low
TCGA-CR-7374	0.0825	0	3.92394	3.78004	2.19626	4.30798	4.56045	8.39388	1.3401	0.79235	low
TCGA-CR-7376	2.66083	1	2.81673	4.05253	2.3809	3.45376	4.0071	7.78136	1.74836	1.12598	high
TCGA-CR-7377	0.76417	0	2.71488	4.47742	2.59614	3.83664	4.20004	7.54718	1.42386	1.0996	high
TCGA-CR-7385	2.72917	0	3.30853	4.34488	2.48997	3.92819	3.573	7.73811	2.00273	0.43085	low
TCGA-CR-7386	3.915	1	2.2946	4.02614	2.07149	3.21597	4.06541	7.83035	0.93845	2.33335	high
TCGA-CR-7389	1.07333	0	2.68371	4.25531	2.3751	3.59734	3.90462	8.10282	0.55566	2.05258	high
TCGA-CR-7390	4.12833	0	3.2713	4.28388	1.7733	3.62239	3.75418	7.43039	0.29753	0.70087	low
TCGA-CR-7392	3.90083	0	2.96627	4.23796	2.12122	3.68941	3.88748	7.67995	1.39485	0.76961	low
TCGA-CR-7394	3.685	0	2.76999	4.84873	1.97907	3.18477	3.85228	7.23671	1.84706	0.47384	low
TCGA-CR-7397	2.06417	0	2.8501	4.68251	2.03036	3.0948	3.52007	7.78609	0.76952	0.94913	low
TCGA-CR-7398	0.42667	0	3.91719	3.89234	1.9544	4.35106	4.55514	7.3457	0.9362	0.46717	low
TCGA-CR-7401	2.94833	0	2.90647	4.37945	1.93535	3.48865	4.07845	7.79121	0.50978	1.26822	high
TCGA-CR-7404	4.03	0	3.7405	4.42751	2.22767	4.08345	4.09482	7.19215	2.10133	0.2307	low
TCGA-CV-5430	11.61	1	3.47408	4.30969	3.04415	3.63382	3.88192	8.37451	1.48502	1.20404	high
TCGA-CV-5434	9.0725	1	3.32618	3.81913	1.95384	4.20135	4.24363	7.48559	1.24099	0.62844	low
TCGA-CV-5435	6.34833	1	3.55254	4.02596	1.51395	3.74052	4.26086	7.52363	0.21923	0.7361	low
TCGA-CV-5436	1.59917	1	3.59465	3.15772	2.28073	4.12136	4.9415	7.83595	0.41105	2.1602	high
TCGA-CV-5439	1.495	0	2.42443	4.87341	2.753	3.76549	4.22354	6.89094	0.68404	1.30857	high
TCGA-CV-5440	8.95167	0	3.78802	3.73577	1.92909	3.71166	4.64653	7.83829	0.21764	1.28813	high
TCGA-CV-5443	7.62167	0	3.45664	4.06037	2.89886	4.80188	4.7339	8.44016	2.22146	0.89078	low
TCGA-CV-5444	6.67167	1	3.34234	3.69634	2.33935	4.07736	4.1922	7.54847	1.28457	0.85238	low
TCGA-CV-5966	1.49167	1	3.07978	3.40318	2.58843	4.31143	5.52986	9.01092	0.51418	6.89266	high
TCGA-CV-5970	1.11167	0	2.75022	4.20878	2.20648	3.33387	3.9611	6.97456	0.43948	1.26045	high
TCGA-CV-5971	1.91917	0	3.46381	4.14072	2.53434	4.05098	4.29562	7.93423	1.29435	0.89784	low
TCGA-CV-5978	0.58833	0	2.7337	3.93529	2.5744	3.68371	4.36048	7.50163	0.48174	2.41515	high
TCGA-CV-5979	3.6	0	3.16635	4.48746	1.52615	3.78096	4.44729	7.10303	0.34558	0.64894	low
TCGA-CV-6003	4.55833	0	2.70417	4.61514	1.99588	3.69577	5.19065	7.73314	0.25057	2.33633	high
TCGA-CV-6441	0.79917	1	3.08699	4.53965	2.4163	3.77095	4.63141	7.65552	0.08574	1.81587	high
TCGA-CV-6934	0.17833	1	3.30484	4.04329	2.26753	3.57351	4.32896	7.26977	1.26004	0.81219	low
TCGA-CV-6936	0.45417	1	3.46718	4.22734	3.15774	3.99317	4.33099	7.75565	1.0408	1.32389	high
TCGA-CV-6938	0.39417	1	3.15655	3.53885	2.21163	4.16866	5.27674	7.62191	0.88383	1.9751	high
TCGA-CV-6941	0.93667	0	3.45791	4.29942	2.18877	4.06013	4.22483	7.50146	1.04093	0.60051	low
TCGA-CV-6943	1.64833	1	2.92581	4.11746	1.89466	3.89775	3.83601	7.77655	1.98052	0.52965	low
TCGA-CV-6948	3.52917	1	3.32439	3.87772	3.03011	3.4211	4.09355	7.53241	0.95928	1.67179	high
TCGA-CV-6950	1.25667	1	2.81429	3.73153	1.61612	4.04558	4.52991	7.95349	0.51043	1.67039	high
TCGA-CV-6952	0.50667	1	2.43065	3.88793	2.08691	2.51868	4.75435	7.60267	0.65168	4.23339	high
TCGA-CV-6953	4.4925	1	2.94115	5.19179	1.82358	3.59998	3.95427	6.58821	0.56396	0.38507	low
TCGA-CV-6954	5.48083	1	3.53842	4.28346	1.92861	3.71252	4.29226	7.75583	0.28457	0.93033	low
TCGA-CV-6959	0.70083	1	3.30457	3.86613	1.89905	3.88389	4.26632	5.53371	0.7121	0.3431	low
TCGA-CV-6962	0.345	1	3.45742	4.06166	2.57748	4.27716	4.24602	8.38057	0.14385	1.82315	high
TCGA-CV-7090	14.3783	0	2.94759	3.72891	1.9307	3.55564	3.91713	7.97436	0.6778	1.53428	high
TCGA-CV-7095	1.56583	1	2.74898	4.76637	1.9814	4.30659	4.26849	8.37201	0.20738	1.47769	high
TCGA-CV-7099	0.665	1	3.28601	4.15435	2.07322	3.5451	4.58101	8.08379	0.73598	1.50257	high
TCGA-CV-7100	0.75	1	2.81681	4.23169	2.48267	3.79354	5.52019	8.54735	0.80996	4.43478	high
TCGA-CV-7103	4.35583	1	3.37686	4.406	1.76505	4.01418	3.70669	6.96754	0.37361	0.39571	low
TCGA-CV-7104	1.07583	1	2.58196	4.33297	2.31018	3.73266	4.15641	8.21672	0.62123	2.24859	high
TCGA-CV-7177	1.815	1	2.94693	4.20466	1.80375	3.55486	4.55683	7.8612	0.21383	1.80539	high
TCGA-CV-7180	0.895	0	3.25023	4.58445	1.73064	4.29141	4.09056	6.42159	0.26385	0.34034	low
TCGA-CV-7242	2.9975	0	3.52175	4.18421	1.92341	3.94729	4.66521	8.01583	1.94094	0.56509	low
TCGA-CV-7245	2.18167	1	3.16652	3.8881	1.69782	4.12761	4.21895	7.49951	0.8894	0.70828	low

TCGA-CV-7248	1.42667	1	3.43027	4.25624	2.16109	3.7166	5.2969	7.93784	1.27326	1.29758	high
TCGA-CV-7250	7.93917	1	2.79052	4.03395	1.97392	3.76023	4.06267	4.16701	1.26391	0.18204	low
TCGA-CV-7252	0.41333	1	3.24716	3.81638	2.45133	3.81594	4.71614	7.79814	0.56728	2.054	high
TCGA-CV-7253	0.98833	1	2.66121	4.16319	2.35016	3.7228	3.07981	6.37786	0.45778	0.62831	low
TCGA-CV-7261	4.13917	1	3.66597	3.88499	2.25949	4.50135	4.59645	8.16356	0.38125	1.24224	high
TCGA-CV-7406	4.785	1	3.30914	4.33056	2.10673	3.86846	4.20902	7.64731	0.14351	1.09185	high
TCGA-CV-7407	2.95917	1	2.47113	3.55679	2.4627	3.08868	4.37746	7.9646	0.78271	4.52271	high
TCGA-CV-7410	17.5675	1	3.11682	3.95134	2.37583	3.91063	4.4101	8.19296	3.0007	0.65896	low
TCGA-CV-7416	2.08917	1	2.79918	3.54949	1.93695	3.47798	3.65579	6.71457	0.16598	1.17258	high
TCGA-CV-7418	2.16	1	3.21664	3.46816	2.83964	3.97992	4.84931	8.13272	0.81254	3.27003	high
TCGA-CV-7422	2.83917	1	3.44858	3.61641	1.88605	4.21784	4.7129	7.8773	0.63392	1.21514	high
TCGA-CV-7424	1.24	1	3.36102	4.01679	2.32281	3.47824	4.65777	7.46579	1.14802	1.1462	high
TCGA-CV-7425	4.70333	1	3.06201	5.10641	1.97273	3.33043	4.11463	7.55095	0.78736	0.68913	low
TCGA-CV-7429	0.29333	1	2.90099	4.29184	2.32119	4.32299	4.85804	7.76515	0.49849	1.74027	high
TCGA-CV-7437	1.385	1	2.85655	3.91873	2.37998	3.753	4.16835	7.68327	0.11796	2.28603	high
TCGA-CV-7446	2.9925	1	2.95548	3.37121	2.6133	2.78879	4.05557	6.85352	0.37162	2.5891	high
TCGA-CV-7568	2.5375	0	2.47292	3.5581	2.21807	3.66104	4.43348	7.21444	0.5344	2.52356	high
TCGA-CV-A45O	2.33	0	2.45384	5.36784	1.82215	2.54164	3.8265	6.43991	0.81909	0.58542	low
TCGA-CV-A45W	3.8275	1	3.65196	3.9632	2.20345	4.38106	4.91716	8.13066	1.78672	0.73096	low
TCGA-CV-A45X	0.54167	1	3.42211	4.81245	1.85298	3.40964	4.50748	7.4255	0.49959	0.71927	low
TCGA-CV-A45Z	4.01333	1	3.77748	3.55926	2.06856	4.60869	4.67266	7.64214	2.13743	0.41077	low
TCGA-CV-A464	4.71417	1	3.38366	4.05766	2.10877	4.20881	5.09501	7.48742	1.13719	0.92398	low
TCGA-CV-A6JD	0.49833	0	3.39964	3.45498	1.98874	3.59944	4.77989	8.5256	1.00966	2.17092	high
TCGA-CV-A6JZ	1.955	0	3.51748	4.09508	1.98907	3.95772	4.84162	8.35135	1.80269	0.83551	low
TCGA-CV-A6K2	0.8675	1	2.8999	3.67793	2.53268	2.65676	4.52645	7.8092	0.63439	4.10132	high
TCGA-CX-7219	2.86083	0	3.50743	3.93412	2.27585	3.12192	4.32351	7.53606	0.95606	1.15405	high
TCGA-D6-6516	2.11583	0	2.56194	4.85429	2.07228	4.42572	4.01286	7.38621	0.78506	0.68604	low
TCGA-D6-6517	0.79917	0	2.88875	4.2057	2.28595	3.53168	4.48482	7.62787	0.67309	1.75207	high
TCGA-D6-6823	1.91917	0	3.70432	4.00871	1.55628	5.09062	4.56811	7.35261	0.59289	0.36477	low
TCGA-D6-6824	0.21083	0	2.99853	4.34801	2.40344	3.92442	3.91216	8.12655	1.00845	1.15387	high
TCGA-D6-6826	0.9525	0	2.9669	3.7251	2.16516	3.37155	4.37196	7.15709	0.89735	1.40806	high
TCGA-D6-8568	2.0775	0	3.30001	3.94518	2.17496	4.38661	4.00827	7.77001	0.68604	0.84726	low
TCGA-D6-8569	2.10833	0	3.11229	3.72064	2.97968	3.28033	5.09767	7.65364	0.84963	3.88101	high
TCGA-D6-A4ZB	1.02917	0	3.40225	4.27018	2.4218	3.81944	4.10809	7.78589	1.25579	0.78176	low
TCGA-D6-A6EK	2.39583	0	2.70163	5.07141	1.32246	4.29492	4.05709	7.29396	1.9805	0.21277	low
TCGA-D6-A6EM	0.635	0	3.02442	3.63488	2.46791	5.26535	5.47524	8.32375	1.85329	1.55784	high
TCGA-D6-A6EN	1.88083	0	3.27574	3.79584	1.78235	4.91427	5.00084	8.18826	2.29366	0.56167	low
TCGA-D6-A6ES	1.065	0	2.9093	4.84348	1.25548	3.52868	4.15549	8.1073	0.29199	0.89707	low
TCGA-DQ-5624	4.8675	1	2.98585	3.46089	2.37302	3.01799	3.98481	7.98848	0.3972	3.19653	high
TCGA-DQ-5625	3.10167	1	2.91205	4.00744	2.06063	3.4709	4.21176	8.10007	1.38138	1.35153	high
TCGA-DQ-5630	2.82	1	2.66279	3.77714	2.3566	2.89375	5.06673	8.32576	1.38138	4.49134	high
TCGA-DQ-5631	1.5	1	2.92767	3.74205	2.01459	3.46686	4.52534	7.5931	1.76665	1.12588	high
TCGA-DQ-7591	1.7025	0	2.63981	3.20607	2.54685	4.15561	5.05835	7.44756	2.40818	1.67854	high
TCGA-F7-7848	3.09667	0	3.46998	4.61938	1.95903	4.66887	3.58683	7.27636	2.20287	0.13633	low
TCGA-F7-8298	2.72417	0	3.51201	3.55659	2.73535	3.8645	3.60062	8.32653	0.48033	1.75879	high
TCGA-F7-8489	1.80167	0	2.90999	3.65796	2.26346	3.17471	4.58049	7.59973	1.83512	1.53107	high
TCGA-F7-A61S	1.57667	0	3.09599	3.66527	2.55099	3.59415	4.76204	8.08345	1.20772	2.47703	high
TCGA-F7-A61W	0.03833	0	3.1197	4.12717	2.48447	4.60042	5.27275	8.02163	0.2286	2.48926	high
TCGA-F7-A620	1.48667	1	3.03131	3.82535	2.39395	4.10931	5.32004	8.17924	1.76662	1.89852	high
TCGA-F7-A623	1.68667	0	3.15556	4.68205	2.48988	4.21382	3.98212	7.1205	0.83336	0.56887	low
TCGA-F7-A624	1.035	0	2.92427	4.44014	2.45486	4.45951	5.22889	8.98314	2.25899	1.55117	high
TCGA-H7-8502	1.25417	0	3.2089	3.77993	2.53248	3.66333	4.65632	7.41812	0.32901	2.15738	high
TCGA-HD-7229	2.81167	0	3.31828	3.58974	1.99486	3.74034	4.40972	7.70529	0.88873	1.22297	high
TCGA-HD-7832	2.28833	0	3.37347	3.63631	2.5641	3.53517	4.74853	7.96855	0.33729	2.98393	high
TCGA-HD-8314	1.83417	0	3.3778	4.25313	2.5787	4.38135	3.51986	7.82939	1.55944	0.47503	low
TCGA-HD-8635	1.9025	0	2.85052	3.49851	2.58574	3.22744	4.07372	7.13969	0.98233	1.90647	high
TCGA-HD-A633	1.1525	0	3.30251	3.98043	2.29972	3.51374	5.03821	7.49109	0.24243	2.18977	high
TCGA-HD-A6I0	0.575	0	2.97688	4.45997	1.23219	4.7174	4.19707	7.14651	1.07734	0.28676	low
TCGA-IQ-7630	1.3275	0	3.3166	3.8821	2.50925	3.91851	4.9705	7.6431	0.62785	1.91655	high
TCGA-IQ-7631	3.20833	0	3.14117	3.62735	1.50956	2.45652	3.51242	7.75869	0.17299	1.56779	high
TCGA-IQ-A61E	3.14	0	3.13319	3.82129	2.24206	4.22556	5.10009	7.90446	1.11486	1.65167	high
TCGA-IQ-A61G	0.98583	0	2.89874	4.03328	3.09149	3.95651	4.78501	7.98909	0.13452	4.5315	high
TCGA-IQ-A61H	3.11583	0	3.28689	3.21103	2.24998	3.41849	5.38004	7.97338	0.57265	4.12814	high
TCGA-IQ-A61J	2.795	0	3.09248	3.96606	2.29046	2.67861	4.30773	7.70352	1.0476	1.88934	high
TCGA-IQ-A61O	1.1525	1	2.49971	3.68917	1.97893	2.83269	4.48606	7.62243	1.04068	2.73865	high
TCGA-IQ-A6SH	1.28917	0	2.89983	4.75898	1.35856	3.90271	5.38972	8.14128	0.7615	1.2753	high
TCGA-KU-A66S	1.11167	1	3.24062	2.8495	2.27026	3.47076	5.06783	8.40529	2.1124	2.61852	high
TCGA-KU-A66T	1.51083	0	3.70682	4.013	1.99613	2.99781	4.26982	7.97477	0.13107	1.51561	high
TCGA-MT-A51W	1.19667	0	2.93957	4.84288	2.025	3.28109	4.2709	7.36643	0.64125	0.94749	low
TCGA-MT-A67D	0.15333	0	3.19966	4.58953	2.06442	4.41806	5.11668	6.60859	0.95646	0.52727	low
TCGA-MZ-A5BI	0.59417	1	3.89629	4.47862	2.55098	4.4658	4.51779	8.80017	2.88835	0.38743	low
TCGA-MZ-A6I9	1.33833	1	3.58677	4.8797	1.97726	4.50205	3.97614	8.49506	0.92764	0.46718	low
TCGA-MZ-A7D7	1.4975	0	2.25291	3.45752	1.87612	2.83024	6.21773	9.07234	0.11257	24.4712	high
TCGA-P3-A5Q5	2.49083	0	3.43523	3.90199	2.43834	4.36164	4.71159	8.03525	2.91327	0.52156	low
TCGA-P3-A6SW	3.06583	0	3.43544	3.85515	2.53677	4.71699	4.3597	7.12086	2.0086	0.40791	low

TCGA-P3-A6SX	3.915	1	2.93913	3.90268	2.06961	3.8701	4.45497	7.24526	0.27678	1.49322	high
TCGA-P3-A6T2	6.29083	0	3.38845	3.49399	2.56383	3.87782	5.43108	7.08311	0.28761	2.56173	high
TCGA-P3-A6T4	0.17	1	2.92283	4.40943	2.37939	3.31067	4.88451	8.00774	0.54829	2.74194	high
TCGA-P3-A6T6	1.08167	1	3.41552	4.70819	2.14798	4.57256	3.86833	8.8647	1.60957	0.51091	low
TCGA-P3-A6T8	1.095	0	2.90007	3.64273	2.63278	3.88248	4.68503	7.39294	0.4671	2.63864	high
TCGA-QK-A6IJ	1.05917	0	2.83813	5.09812	1.07581	4.10131	4.47857	6.4493	1.92236	0.14824	low
TCGA-QK-A8Z8	0.46833	1	2.50756	4.28197	2.39248	4.05395	4.95115	7.04855	0.94501	1.61204	high
TCGA-QK-A8Z9	1.22917	1	3.04396	3.62879	1.79791	3.04597	3.92772	8.07123	1.19772	1.39761	high
TCGA-QK-A8ZA	1.01583	1	3.07231	3.55484	2.5471	2.71196	4.0055	7.07842	0.24106	2.49548	high
TCGA-QK-A8ZB	1.48417	0	2.86603	4.6396	2.07345	3.72016	4.61446	7.94462	0.46478	1.62179	high
TCGA-QK-AA3K	0.6925	0	3.15068	3.96687	2.23098	4.11784	4.52849	8.58107	1.77466	1.22315	high
TCGA-RS-A6TO	1.05917	1	2.51293	4.41405	2.05452	4.70652	4.24655	7.66601	1.06718	0.87792	low
TCGA-T2-A6X2	2.70167	0	3.3637	4.00559	2.12544	3.67608	4.02422	7.30729	1.23679	0.62472	low
TCGA-T3-A92M	1.14167	0	3.70208	4.35939	2.73119	3.48462	4.69635	8.23576	1.69116	1.10262	high
TCGA-TN-A7HJ	1.10333	0	2.70616	4.33205	1.96189	3.41134	4.23865	7.25144	0.51214	1.30269	high
TCGA-TN-A7HL	1.695	0	3.7002	4.00172	2.06649	5.45746	4.99916	8.22828	3.07197	0.2545	low
TCGA-UF-A71A	0.23583	1	2.56672	3.94133	2.56263	3.70408	4.28614	7.73964	0.13474	3.40646	high
TCGA-UF-A71B	4.1225	0	3.4038	4.13936	2.1719	3.87842	4.9646	8.35876	0.73644	1.7774	high
TCGA-UF-A71D	4	0	3.46118	4.14225	1.94215	4.32669	3.89147	7.16295	2.2049	0.21332	low
TCGA-UF-A71E	4.1175	1	3.32757	4.42118	2.04455	4.72064	4.16183	8.12486	0.1926	0.86479	low
TCGA-UF-A7J9	3.7175	0	3.46998	3.74027	2.0604	4.25668	4.29731	7.58507	0.59791	0.88968	low
TCGA-UF-A7JD	2.02333	1	3.21566	4.34615	2.49492	4.07486	4.16622	8.06411	1.29538	0.94233	low
TCGA-UF-A7JK	1.16083	1	2.72078	3.62945	2.29808	3.44213	4.51426	8.49975	0.09841	5.50138	high
TCGA-UF-A7JT	2.71833	1	3.23662	3.93712	2.14649	4.03709	4.87891	8.57615	2.24283	1.08628	high
TCGA-WA-A7GZ	1.71083	1	2.79436	4.35158	2.1726	4.08088	4.97556	8.0366	0.21824	2.52389	high
TCGA-WA-A7H4	1.2125	0	3.43054	3.60026	2.39798	3.78336	4.51202	8.0312	1.03247	1.6042	high

Supplementary Table S3. The riskscores of HNSCC patients in the GSE65858.

ID	futime	fustat	MAP2K7	MAPK3	MAPK9	ORAI1	PSMA1	UBB	ZAP70	Riskscore	Risk
GSM1607684	3.0137	0	6.8378	8.6839	8.0469	7.2976	7.0059	9.9392	6.4449	0.05447	high
GSM1607685	5.0274	0	6.8458	9.1742	7.9355	6.9773	6.9476	9.926	6.4687	0.04325	low
GSM1607686	6.55616	1	6.7241	9.0282	7.5524	6.8338	6.8792	9.3996	6.4231	0.03284	low
GSM1607687	3.32055	0	6.7412	8.5502	7.8998	7.0568	6.9481	9.5511	6.3596	0.05261	high
GSM1607688	2.70411	1	6.6641	8.9882	8.1697	6.8932	6.9602	9.7558	6.4397	0.05995	high
GSM1607689	5.31233	1	6.7602	9.7611	7.6796	6.7497	6.6524	9.2661	6.4267	0.02096	low
GSM1607690	3.6411	0	6.7309	9.1626	7.8929	7.0043	6.7957	9.7105	6.496	0.03775	low
GSM1607691	2.57808	0	6.7316	8.974	8.3991	7.2531	7.2833	10.0738	6.3909	0.07969	high
GSM1607692	3.38082	0	6.7282	8.9496	8.125	7.0716	7.4806	10.2458	6.353	0.08978	high
GSM1607693	2.80548	0	6.7055	8.7181	8.3008	6.8447	6.948	9.5205	6.4195	0.06529	high
GSM1607694	2.42192	0	7.2819	9.0292	7.788	6.9304	7.0676	10.1893	6.3926	0.03942	low
GSM1607695	2.8	0	6.7872	9.3454	8.1116	6.7262	7.0493	9.8956	6.3993	0.05409	high
GSM1607696	2.93973	1	6.746	8.8226	8.2788	6.9201	7.3137	9.6986	6.4163	0.07516	high
GSM1607697	3.53151	0	6.7821	9.0589	7.903	7.5196	6.865	9.68	6.524	0.03177	low
GSM1607698	2.92055	1	6.7686	9.0091	7.7192	7.0212	7.1317	9.5011	6.5353	0.03702	low
GSM1607699	2.84384	0	6.7907	9.4871	7.8614	7.1863	6.8539	9.5345	6.4904	0.0268	low
GSM1607700	1.5726	1	6.7883	9.5825	8.1942	6.9425	6.9007	9.5043	6.3519	0.03669	low
GSM1607701	6.13699	1	7.3239	8.7548	7.99	6.7692	6.7628	10.4469	6.4388	0.05007	high
GSM1607702	4.89315	0	6.7707	8.6609	7.8464	7.0389	7.0632	8.8609	6.5289	0.03333	low
GSM1607703	4.99178	0	6.7496	9.7259	7.5479	7.0159	6.6806	9.7882	6.4367	0.02334	low
GSM1607704	1.63014	1	6.7916	9.5019	8.0806	6.9068	6.8938	10.0148	6.3784	0.04551	low
GSM1607705	2.74795	0	6.5331	9.0684	8.1628	6.8662	6.9951	9.7845	6.4821	0.06449	high
GSM1607706	2.89863	0	6.7416	8.3891	8.3546	7.241	7.0123	10.2514	6.411	0.09752	high
GSM1607707	3.14795	0	6.8669	9.217	7.6892	7.2699	7.2131	9.7082	6.5292	0.03211	low
GSM1607708	3.42192	0	6.9495	8.9325	8.104	6.7781	6.8957	9.5348	6.4674	0.04337	low
GSM1607709	3.93151	0	6.7896	9.1949	7.8561	7.0116	6.8137	9.2956	6.4654	0.02911	low
GSM1607710	3.43562	0	6.6827	9.2343	8.3002	6.9272	7.3339	9.7624	6.4512	0.06669	high
GSM1607711	2.91507	0	6.638	8.793	8.2744	7.5057	6.9139	10.2908	6.4161	0.07234	high
GSM1607712	4.72329	1	7.0318	9.8858	7.7704	6.8017	6.6751	9.9098	6.3503	0.02396	low
GSM1607713	4.62192	0	6.7617	9.5868	7.8547	7.3475	6.572	9.3	6.4119	0.01977	low
GSM1607714	1.29589	1	6.9159	9.1993	7.8676	7.3213	7.0606	9.4713	6.3669	0.0304	low
GSM1607715	3.32603	1	6.7386	8.7804	8.0179	7.2164	7.1069	10.0293	6.3686	0.06433	high
GSM1607716	2.1726	1	6.7879	9.2397	7.5956	7.1805	6.7747	9.4806	6.3442	0.0261	low
GSM1607717	2.65205	1	6.9833	9.864	7.8286	6.8387	6.5804	9.3272	6.4597	0.01753	low
GSM1607718	2.59452	0	6.8493	8.766	8.0182	6.9412	7.1648	9.1142	6.4834	0.0416	low
GSM1607719	2.55616	0	6.7627	8.8445	8.0937	7.2338	6.8717	10.425	6.4265	0.06704	high
GSM1607720	2.42466	0	6.95	9.4009	8.0341	6.9147	6.6607	10.0644	6.4502	0.03667	low
GSM1607721	2.42192	0	6.7358	8.2871	8.434	7.0864	7.1251	9.606	6.4227	0.0878	high
GSM1607722	2.56712	0	6.734	9.2722	8.0648	6.9837	6.939	9.6929	6.4737	0.04268	low
GSM1607723	3.00822	1	6.7491	8.8267	7.8181	6.6994	6.9833	9.0626	6.5096	0.03746	low
GSM1607724	2.74521	1	6.8102	9.1599	8.1288	7.1937	6.749	9.6767	6.3197	0.03977	low
GSM1607725	4.13151	0	6.8526	9.3318	8.4065	6.9053	7.4569	9.5333	6.5252	0.05535	high
GSM1607726	3.26301	1	6.7746	9.0009	7.8374	7.1721	6.7536	9.864	6.4964	0.03792	low
GSM1607727	3.11507	0	6.6432	9.4508	7.8742	7.1335	6.8914	10.0005	6.3747	0.04197	low
GSM1607728	3.50959	1	6.638	9.8469	7.6536	6.8831	7.1137	9.7091	6.3999	0.03213	low
GSM1607729	2.06301	1	6.9173	8.9516	8.0079	6.8009	7.0046	9.8339	6.4343	0.05096	high
GSM1607730	2.88219	0	6.8517	8.4648	8.0638	6.9661	6.9484	9.5747	6.4187	0.05675	high
GSM1607731	3.15068	1	6.7681	9.9244	8.098	6.7403	7.1029	9.6796	6.3193	0.03922	low
GSM1607732	3.47945	1	6.742	9.0034	8.2994	6.7795	7.4061	9.6778	6.4595	0.07478	high
GSM1607733	5.5589	0	6.8692	9.1106	7.5306	7.2125	6.7328	9.5016	6.4381	0.02362	low
GSM1607734	0.73151	1	7.1487	9.1073	8.1524	7.3044	6.8158	9.3268	6.4064	0.026	low
GSM1607735	3.33973	0	6.7858	9.2307	7.8952	6.9486	6.6947	9.5199	6.4203	0.03233	low
GSM1607736	2.94247	0	6.5986	9.4067	8.1999	6.6704	6.995	10.2739	6.4593	0.07363	high
GSM1607737	3.23014	1	6.6483	8.9306	7.71	6.9139	6.9814	10.2038	6.3435	0.06213	high
GSM1607738	2.92055	1	6.7502	9.1272	7.9384	7.1675	6.9664	9.4737	6.4482	0.03614	low
GSM1607739	4.70411	0	6.7296	8.9013	8.0447	6.951	7.03	9.7844	6.3604	0.059	high
GSM1607740	5.6411	0	6.8367	9.313	8.2357	7.2393	7.3095	9.9325	6.5416	0.05053	high
GSM1607741	0.48493	1	6.8305	8.9873	7.8788	6.7988	6.9599	9.9179	6.5155	0.04861	low
GSM1607742	0.75068	1	6.8794	8.6272	7.5721	7.078	7.4078	10.0344	6.4115	0.05776	high
GSM1607743	5.37534	1	6.7495	8.9317	7.7322	7.1435	6.8693	9.5874	6.3859	0.03695	low
GSM1607744	5.48219	0	7.1049	9.1096	8.171	6.8573	6.7076	9.4226	6.4802	0.03089	low
GSM1607745	4.37534	0	6.7731	9.5638	8.329	7.396	6.8202	9.1112	6.345	0.02717	low
GSM1607746	2.58904	0	6.7413	8.9828	8.039	7.1284	7.2771	9.6497	6.4142	0.05386	high
GSM1607747	2.66849	0	6.71	9.5591	8.0025	7.0405	7.1634	9.901	6.4204	0.04501	low
GSM1607748	2.74521	0	6.7303	8.8722	8.315	6.9309	6.7882	9.509	6.4054	0.05387	high
GSM1607749	2.78356	0	6.7878	8.8959	8.2493	6.9547	7.0836	10.3806	6.431	0.0845	high
GSM1607750	2.75068	0	6.7592	8.5573	8.3946	6.5976	7.251	10.3566	6.4636	0.13366	high
GSM1607751	2.74521	1	6.6875	8.891	7.8463	6.8904	7.3073	9.7615	6.3434	0.06343	high
GSM1607752	2.69041	0	6.6043	9.7232	8.0668	7.3088	6.9267	9.9385	6.5246	0.03643	low
GSM1607753	2.63288	0	7.0832	9.3725	8.2155	6.9402	7.1106	9.2132	6.449	0.03053	low
GSM1607754	2.6137	0	6.7507	9.0444	7.8496	7.0614	7.3111	10.0057	6.3657	0.05892	high
GSM1607755	2.60548	1	6.8358	8.9725	7.9605	6.8601	7.3485	9.4579	6.4907	0.0487	low

GSM1607756	2.5863	0	6.7475	9.4086	8.2958	6.8962	6.7356	9.8478	6.337	0.04877	low
GSM1607757	2.57808	0	6.7272	8.8947	7.9173	7.0882	7.1622	10.2325	6.4271	0.06701	high
GSM1607758	2.48219	1	6.6131	9.3021	8.042	7.0442	6.7862	9.6526	6.4088	0.0416	low
GSM1607759	2.53973	0	6.8906	9.2127	8.1179	6.7568	7.0118	9.6896	6.2985	0.04948	low
GSM1607760	2.45479	0	6.7359	8.1924	7.8188	6.9504	7.2375	9.7128	6.4803	0.07324	high
GSM1607761	2.47397	1	6.7286	9.148	7.6821	6.6559	6.9409	10.4978	6.4243	0.06241	high
GSM1607762	2.39726	0	6.847	9.2501	7.8247	6.9782	6.799	9.4916	6.4004	0.03043	low
GSM1607763	2.48219	0	6.7686	8.8249	8.1422	7.1244	6.9656	9.4998	6.4578	0.04762	low
GSM1607764	4.95342	1	6.6998	9.5139	8.1219	7.3909	6.6698	9.719	6.469	0.03074	low
GSM1607765	5.03288	0	6.7469	9.6357	8.1793	7.3181	6.9019	9.7001	6.3902	0.03419	low
GSM1607766	0.83288	1	6.7063	9.3554	7.8266	6.6009	7.1421	9.4548	6.3502	0.04383	low
GSM1607767	1.7863	1	6.7045	9.3595	7.7746	7.136	7.005	9.7119	6.3779	0.03628	low
GSM1607768	5.56986	1	6.8869	9.6147	7.5373	7.2254	6.5661	8.9364	6.569	0.01207	low
GSM1607769	5.00548	0	6.7866	9.0826	8.1986	6.7752	7.1165	9.6015	6.4421	0.05571	high
GSM1607770	5.12877	1	6.9418	9.5289	7.8623	7.1578	6.8185	10.0562	6.3816	0.0317	low
GSM1607771	5.54795	0	6.7633	9.2063	7.9228	6.8593	6.9297	10.0048	6.524	0.04711	low
GSM1607772	5.08219	0	7.1173	8.7586	7.938	7.2623	6.8831	9.4192	6.497	0.02924	low
GSM1607773	6.32877	0	6.8056	9.1886	8.0642	6.8444	6.7273	9.5267	6.4527	0.03748	low
GSM1607774	1.36712	1	6.6761	8.4124	8.0746	6.9254	7.2878	10.1046	6.3866	0.10413	high
GSM1607775	1.86027	1	6.6365	8.6528	8.438	7.003	7.1421	10.1759	6.4379	0.10784	high
GSM1607776	3.01096	0	6.8049	9.1933	7.6222	6.7121	6.4813	8.9558	6.4006	0.02104	low
GSM1607777	3.33973	0	6.7862	8.7829	8.4071	6.7202	7.0179	9.7446	6.4041	0.07728	high
GSM1607778	5.21644	0	6.8664	8.7302	7.5928	6.7016	6.8648	9.7422	6.3405	0.04489	low
GSM1607779	0.06301	1	6.7097	8.7154	8.1836	7.2438	7.09	9.5879	6.4716	0.05642	high
GSM1607780	3.22192	0	6.8076	9.1912	7.8538	7.2357	7.2672	10.5801	6.5932	0.05746	high
GSM1607781	3.14247	0	6.5967	9.5136	7.7695	7.0275	7.0125	9.9675	6.4733	0.04109	low
GSM1607782	3.2274	0	6.9226	8.8789	8.0822	7.1263	6.8679	9.694	6.5335	0.04055	low
GSM1607783	3.07671	0	6.6654	8.7749	8.08	6.861	7.0246	9.9201	6.4532	0.07081	high
GSM1607784	2.87945	0	6.7693	9.0448	7.7598	7.073	6.7572	9.6408	6.4179	0.0345	low
GSM1607785	3.24384	0	6.9237	9.0626	7.8498	6.8638	7.0147	9.1239	6.4238	0.03067	low
GSM1607786	3.26575	0	6.7572	9.2303	8.1021	7.046	7.3922	9.6528	6.4123	0.05354	high
GSM1607787	4.95342	0	6.9429	9.2231	7.7981	7.3972	7.2357	9.6663	6.3781	0.03268	low
GSM1607788	3.64932	0	6.6519	9.0281	8.2027	7.3066	6.9	9.6695	6.3801	0.04948	high
GSM1607789	3.57534	0	6.801	8.8727	8.1246	7.0849	6.9096	9.8908	6.4154	0.0548	high
GSM1607790	2.80548	0	6.6834	8.556	8.1547	6.6617	7.3109	10.1372	6.4374	0.11232	high
GSM1607791	2.90411	0	6.7365	9.0643	7.5397	7.2152	6.9454	9.4669	6.354	0.03022	low
GSM1607792	1.64932	1	6.7199	9.7681	8.2184	6.7423	7.1516	9.7399	6.522	0.04502	low
GSM1607793	2.27671	1	6.722	9.2223	8.1999	6.9562	7.1236	10.157	6.3891	0.06854	high
GSM1607794	2.92329	0	6.7205	9.151	7.7617	6.9911	6.8408	9.558	6.4518	0.03451	low
GSM1607795	0.32329	1	7.1617	9.458	7.7161	7.1135	6.4795	9.0726	6.5121	0.01301	low
GSM1607796	3.03288	0	6.6737	8.2721	8.2714	6.8227	7.1946	9.4362	6.4048	0.08937	high
GSM1607797	2.6137	0	6.7588	8.8654	7.873	6.9378	7.4305	9.7031	6.4457	0.05981	high
GSM1607798	2.28493	0	6.712	9.2778	7.897	7.0042	7.0379	10.0116	6.4672	0.04775	low
GSM1607799	0.69315	1	6.7425	9.7103	8.259	6.6257	6.9993	9.7524	6.3198	0.05011	high
GSM1607800	3.43014	0	6.5474	9.9358	7.8872	6.9419	7.0622	10.2255	6.3439	0.04731	low
GSM1607801	1.4274	1	6.7513	8.5159	8.1595	6.8368	7.2399	10.0656	6.4733	0.09392	high
GSM1607802	2.65205	0	6.729	8.6208	8.2427	7.0046	7.3002	9.9297	6.5279	0.08391	high
GSM1607803	1.17534	1	6.6672	8.9545	7.9988	6.7697	7.192	9.5309	6.4556	0.05739	high
GSM1607804	3.31781	0	6.8615	9.0672	8.4712	6.8572	7.0877	8.7286	6.4777	0.0384	low
GSM1607805	3.23836	0	6.6773	8.9949	7.7083	7.1681	6.7601	9.5386	6.4889	0.0326	low
GSM1607806	1.59452	1	6.8842	8.6058	7.6533	6.8728	6.817	9.6481	6.4147	0.04121	low
GSM1607807	2.72877	0	7.1147	9.23	7.6628	7.2541	6.5459	9.5037	6.4232	0.0183	low
GSM1607808	2.68219	0	6.9812	9.1592	8.2756	7.0316	7.069	9.6591	6.4243	0.04483	low
GSM1607809	2.85753	0	6.7311	9.9193	7.9726	6.9263	7.3313	10.0765	6.388	0.04576	low
GSM1607810	3.47945	1	6.568	8.8798	8.4784	7.1015	6.834	10.0588	6.447	0.08131	high
GSM1607811	2.42466	0	6.7297	9.1458	7.788	6.805	7.0243	9.5908	6.3529	0.04351	low
GSM1607812	3.24658	0	6.6798	8.7287	7.8631	6.7171	7.3742	9.8392	6.3259	0.08045	high
GSM1607813	2.22192	1	7.035	9.758	7.6166	7.1385	6.7742	9.0468	6.4643	0.0134	low
GSM1607814	2.80548	0	6.68	9.0101	8.2232	7.1139	7.033	10.1353	6.4688	0.06824	high
GSM1607815	2.34521	0	6.8455	9.3922	7.5525	6.585	6.8824	9.2486	6.5494	0.02435	low
GSM1607816	2.7863	0	6.843	9.2472	8.0775	7.0439	6.8578	9.8225	6.4974	0.03982	low
GSM1607817	1.33973	1	6.7388	9.0887	8.1742	7.2213	6.8733	10.1282	6.4273	0.0553	high
GSM1607818	2.89589	0	6.644	8.8462	8.2426	7.0908	6.8278	9.8331	6.4693	0.06059	high
GSM1607819	2.89589	1	6.9718	8.6251	7.8857	7.0299	6.8675	9.8652	6.4723	0.04576	low
GSM1607820	2.76438	1	6.7095	8.7655	8.0343	6.6675	7.4685	9.6267	6.4825	0.07642	high
GSM1607821	3.03288	0	6.6672	9.595	7.7942	7.1463	6.8832	9.7893	6.3846	0.03271	low
GSM1607822	2.39726	0	7.0758	9.2762	8.5222	6.8674	7.0558	9.8137	6.4836	0.05064	high
GSM1607823	1.88767	1	6.5945	8.9345	8.0044	6.9042	6.9332	9.7495	6.4935	0.05618	high
GSM1607824	0.75342	1	6.6186	9.1447	7.9155	6.9403	6.9862	10.0615	6.4049	0.05806	high
GSM1607825	1.72877	1	6.7726	8.7874	8.0608	7.0642	7.0567	9.7934	6.5481	0.05447	high
GSM1607826	3.73973	0	6.8135	9.2267	7.9112	7.2851	6.7782	9.6746	6.402	0.03187	low
GSM1607827	2.23288	0	6.6653	8.8114	8.0817	7.174	6.8076	9.6653	6.4241	0.04993	high
GSM1607828	2.1726	0	6.6752	9.1624	8.5402	6.8405	7.2951	9.8993	6.4098	0.08821	high
GSM1607829	0.97534	1	6.9871	8.6437	7.7734	7.1881	7.2881	10.3409	6.3134	0.06551	high
GSM1607830	2.17534	0	6.8215	8.5693	7.9245	7.2203	7.0293	9.9706	6.5362	0.05499	high

GSM1607831	2.1726	0	6.7401	9.297	8.2486	6.8401	7.1787	10.1196	6.5015	0.06714	high
GSM1607832	0.74521	1	6.6405	8.9455	7.7338	6.8051	6.8227	9.4198	6.4337	0.0399	low
GSM1607833	2.80822	0	6.7321	9.6205	8.1538	6.9316	7.2041	9.601	6.4552	0.04247	low
GSM1607834	2.01096	0	6.7548	9.0608	8.1029	7.2392	6.8115	9.9721	6.4042	0.04805	low
GSM1607835	2.31781	0	6.6722	9.1886	8.4286	7.1117	7.3446	9.1015	6.4752	0.04981	high
GSM1607836	1.95342	0	6.681	8.6882	7.6301	7.6526	7.3734	10.4912	6.3797	0.06692	high
GSM1607837	3.41096	0	6.9894	9.6433	7.9133	6.9169	6.7263	10.0934	6.5133	0.03	low
GSM1607838	1.9863	1	6.8097	8.5858	8.3496	6.8586	7.2383	10.1953	6.5374	0.09956	high
GSM1607839	2.06849	0	6.6904	8.5337	8.0845	6.9163	7.0179	9.955	6.3563	0.08136	high
GSM1607840	2.07671	0	6.7079	9.5737	8.1018	6.8387	6.9892	9.8699	6.3608	0.04773	low
GSM1607841	1.14247	1	6.7059	8.7814	7.8652	7.1587	6.8841	9.8378	6.4021	0.0497	high
GSM1607842	3.53699	0	6.7766	9.2075	7.9993	6.8988	6.792	10.0089	6.4491	0.04666	low
GSM1607843	0.73699	1	6.69	8.4951	8.3367	7.1087	7.1255	10.1395	6.4092	0.10017	high
GSM1607844	2	0	6.9713	9.1721	7.8721	6.8519	6.6783	9.9611	6.4883	0.03544	low
GSM1607845	2.41644	0	6.8802	8.8931	7.6147	7.5616	6.9448	9.8559	6.4301	0.03154	low
GSM1607846	2.09315	0	6.6111	9.3832	8.3046	6.845	7.1927	9.7345	6.5261	0.06022	high
GSM1607847	2.90137	0	6.7769	9.6145	7.9445	6.9611	6.7963	9.182	6.4476	0.02436	low
GSM1607848	2.03562	0	6.9753	9.3164	7.6566	6.7502	6.7903	9.1662	6.5057	0.02153	low
GSM1607849	0.70411	1	6.8252	8.9656	8.0037	6.9406	6.9539	9.7688	6.4624	0.04777	low
GSM1607850	1.69041	1	6.494	8.7519	7.8939	6.6509	7.0266	10.4272	6.3624	0.10515	high
GSM1607851	0.32877	1	6.6774	8.9872	8.2351	6.9628	6.9862	10.0793	6.496	0.06931	high
GSM1607852	3.18356	0	6.7402	9.5963	8.1147	7.0149	7.1807	9.5453	6.3669	0.04057	low
GSM1607853	2.07671	0	6.7752	9.1723	8.0115	6.9293	7.0985	9.7829	6.5312	0.04723	low
GSM1607854	2.09589	0	6.7127	9.503	8.329	6.8236	7.3079	9.6991	6.4309	0.05862	high
GSM1607855	0.4	1	6.6437	9.4207	7.8788	6.6613	7.0428	10.2031	6.4745	0.05786	high
GSM1607856	0.5726	1	6.6507	8.9818	8.0947	6.9705	7.1419	9.7806	6.4302	0.06257	high
GSM1607857	2.10685	0	6.6329	8.5814	8.1223	7.1083	7.3833	10.3703	6.3901	0.11277	high
GSM1607858	2.15068	0	6.6246	9.6121	8.1918	7.0681	7.2276	9.2407	6.4568	0.03796	low
GSM1607859	2.30959	0	6.7159	8.6249	7.8431	6.6774	7.1513	10.056	6.4431	0.07821	high
GSM1607860	2.26301	0	6.8277	9.3292	8.1142	6.7237	7.072	8.8162	6.5376	0.02985	low
GSM1607861	0.70685	1	6.6975	8.7466	8.246	7.1588	6.8969	9.9856	6.4079	0.06838	high
GSM1607862	0.38082	1	7.1175	9.346	7.5231	7.3216	6.5738	9.5971	6.5108	0.01579	low
GSM1607863	1.94521	0	6.7111	9.1887	7.9344	7.1398	6.8509	10.0129	6.4124	0.04545	low
GSM1607864	2.10685	0	7.183	9.0422	8.2317	7.3942	7.2885	10.1945	6.3621	0.05153	high
GSM1607865	0.83562	1	6.8375	8.4705	8.1323	7.0292	7.1444	10.5122	6.4162	0.10047	high
GSM1607866	2.3863	0	6.7432	8.9495	7.865	7.0489	7.6269	9.8902	6.5704	0.06286	high
GSM1607867	1.27671	1	6.5959	9.4401	7.6187	7.0603	6.8717	9.6554	6.4588	0.0312	low
GSM1607868	2.34247	0	6.7734	9.2446	7.9951	7.0382	6.9057	9.6074	6.5785	0.03557	low
GSM1607869	2.34795	0	6.7275	8.3868	8.2343	6.6652	7.2077	9.8542	6.5197	0.09869	high
GSM1607870	2.30137	0	6.7791	8.9675	8.0553	7.1463	6.8536	9.4047	6.3853	0.03894	low
GSM1607871	0.40274	1	6.7663	8.9521	8.0149	7.0714	6.7615	9.8484	6.4387	0.04605	low
GSM1607872	0.49863	1	6.7378	9.3994	7.7529	7.3035	6.9012	9.1909	6.3989	0.02357	low
GSM1607873	2.26027	0	6.7119	9.2195	8.1158	6.844	6.9971	9.7014	6.4806	0.04974	high
GSM1607874	2.16438	0	6.6289	9.3106	8.0988	6.9065	7.2219	9.5798	6.5195	0.05037	high
GSM1607875	2.40274	0	6.5755	9.1702	8.1347	7.0635	7.0176	9.5064	6.5507	0.04649	low
GSM1607876	2.2274	0	6.8199	9.181	8.4311	7.0633	6.951	9.5114	6.4752	0.04632	low
GSM1607877	0.73425	1	6.699	9.1986	8.1674	6.7996	7.0826	9.6926	6.4366	0.05627	high
GSM1607878	2.15068	0	6.781	8.9597	7.777	7.0992	6.705	10.3534	6.4877	0.04751	low
GSM1607879	2.12877	0	6.614	8.968	8.2412	7.1893	7.2308	10.2019	6.3818	0.08475	high
GSM1607880	2.13151	0	6.6766	9.0004	7.8119	7.0495	6.9168	9.4326	6.4116	0.03836	low
GSM1607881	2.15616	0	6.8315	9.663	7.8061	7.0698	6.8836	9.4735	6.3705	0.02519	low
GSM1607882	2.05479	0	6.7422	9.2219	8.1668	6.7731	7.0091	9.7766	6.4289	0.05496	high
GSM1607883	0.88767	1	6.7768	8.1568	8.1242	6.6698	7.2851	9.8437	6.3673	0.11046	high
GSM1607884	3.05479	0	6.7878	9.1319	7.7573	7.0779	7.4544	9.9865	6.5155	0.05124	high
GSM1607885	0.76438	1	6.8371	9.238	8.0735	7.0421	6.9371	9.6549	6.4357	0.03953	low
GSM1607886	2.03014	0	6.7068	9.4285	8.3585	7.1323	6.9673	9.384	6.4686	0.03957	low
GSM1607887	2.09315	0	6.62	8.7428	8.147	6.7781	6.8451	9.6059	6.4553	0.06282	high
GSM1607888	2.10685	0	7.0562	9.038	7.9802	6.7171	6.739	9.2893	6.4585	0.03007	low
GSM1607889	2.05753	0	6.7659	9.912	7.9067	6.7465	7.0247	10.3314	6.4536	0.04376	low
GSM1607890	0.58082	1	6.5631	9.5647	7.9022	7.0072	7.4444	10.3131	6.4629	0.06525	high
GSM1607891	0.89041	1	6.8485	9.0847	7.9637	7.1065	6.6867	10.0765	6.4539	0.0417	low
GSM1607892	1.68767	1	6.701	8.8998	8.1927	7.1279	7.0308	9.7722	6.3071	0.06259	high
GSM1607893	2.12603	0	6.9658	8.9509	7.6684	7.2417	6.8828	9.3033	6.4532	0.02471	low
GSM1607894	2.26027	0	6.7562	9.0549	8.0274	6.735	6.8714	9.8066	6.4392	0.05219	high
GSM1607895	3.71781	0	6.6928	8.6544	8.4806	6.9771	7.1798	10.1577	6.5978	0.10036	high
GSM1607896	2.91233	1	6.7788	8.6864	7.9361	7.2074	7.1	9.9598	6.5302	0.05594	high
GSM1607897	0.80548	1	7.0153	9.6559	7.7501	7.2599	6.7185	9.5205	6.6964	0.01619	low
GSM1607898	1.86301	0	6.629	9.0129	7.5774	6.7901	7.1201	9.6972	6.4539	0.04669	low
GSM1607899	1.88767	0	6.5812	9.7119	7.9439	6.7482	6.8211	10.0308	6.4056	0.04514	low
GSM1607900	1.8	0	6.935	8.7858	8.2334	7.0873	7.3741	10.0263	6.5243	0.06995	high
GSM1607901	1.00274	1	6.8095	8.8781	7.7678	7.0157	6.8158	10.2223	6.4161	0.05087	high
GSM1607902	1.67397	1	6.8031	9.084	8.1839	7.4658	6.7102	9.401	6.4688	0.03094	low
GSM1607903	1.82192	0	6.6562	8.7409	8.4934	7.0287	6.9717	9.5372	6.4075	0.07153	high
GSM1607904	1.92055	0	6.5429	9.7804	7.8941	6.7851	6.8276	9.8307	6.4774	0.03787	low
GSM1607905	1.8	0	6.7048	9.1911	8.12	6.932	6.9802	9.9667	6.4333	0.05664	high

GSM1607906	1.73151	0	6.7736	8.9548	8.0527	7.0764	7.3727	9.9413	6.4446	0.06516	high
GSM1607907	0.4411	1	6.88	8.7603	8.1132	7.2006	7.0891	9.5958	6.4602	0.04805	low
GSM1607908	1.13699	1	6.7591	8.7999	8.1702	6.787	7.0469	9.5283	6.4647	0.05895	high
GSM1607909	1.69589	0	6.7421	8.664	7.9497	7.2976	6.9875	10.3253	6.5081	0.06462	high
GSM1607910	1.65753	0	6.7206	8.6333	8.4346	6.88	7.3325	10.2133	6.6272	0.10943	high
GSM1607911	1.7863	0	6.785	8.5912	7.6618	6.9386	7.1699	9.5876	6.4071	0.05039	high
GSM1607912	1.76164	0	6.7854	8.8869	8.0951	6.7235	7.3052	9.711	6.4584	0.0674	high
GSM1607913	1.65205	0	6.8394	9.388	7.7861	7.151	6.9576	9.3129	6.4985	0.02476	low
GSM1607914	3.31507	1	6.824	9.045	7.7638	6.8412	6.9186	9.7784	6.4838	0.04066	low
GSM1607915	1.39726	1	6.6919	8.6482	7.9352	7.0584	7.075	10.1117	6.4534	0.07075	high
GSM1607916	1.8137	1	6.5314	9.5359	7.9423	6.8932	7.3864	10.0395	6.335	0.06545	high
GSM1607917	0.51507	1	6.6631	8.368	7.6756	6.8811	7.0036	9.847	6.4971	0.06344	high
GSM1607918	0.12603	1	6.8946	8.8533	8.3118	7.1018	6.8639	10.1953	6.4252	0.06463	high
GSM1607919	2.14521	0	6.7311	9.2138	8.1313	6.6854	6.9411	9.8432	6.3714	0.05786	high
GSM1607920	2.92329	0	6.7553	8.7061	7.9936	7.263	7.1336	9.9014	6.4668	0.05804	high
GSM1607921	0.94247	1	6.758	9.4984	7.9342	7.2165	7.4066	10.2186	6.5351	0.04995	high
GSM1607922	2.28219	0	6.8044	9.1449	8.2219	6.7012	6.9657	9.5777	6.3991	0.05222	high
GSM1607923	2.20548	0	7.0047	9.4668	8.0548	7.0946	6.8374	9.2573	6.5482	0.0227	low
GSM1607924	0.42192	1	6.6831	9.4104	8.0638	6.9212	7.0548	9.6673	6.3543	0.04677	low
GSM1607925	2.01096	0	6.8966	8.5352	7.8486	6.7641	7.1469	10.5431	6.4244	0.08864	high
GSM1607926	0.54247	1	6.6232	8.6753	7.9851	7.0536	7.0642	10.2047	6.5323	0.07592	high
GSM1607927	1.58356	1	6.9291	8.6021	8.3283	7.2472	7.0647	9.9131	6.5258	0.0623	high
GSM1607928	0.94795	1	6.6561	9.2077	8.226	6.8556	6.7577	9.7024	6.4009	0.0512	high
GSM1607929	1.63288	0	6.717	9.1079	8.2978	7.2338	6.7911	10.0345	6.399	0.05541	high
GSM1607930	1.61644	0	6.9391	9.9628	8.0015	6.8437	6.7753	9.5514	6.573	0.0221	low
GSM1607931	1.63288	0	6.7263	8.9494	7.9361	7.1917	7.549	9.3916	6.4894	0.04937	low
GSM1607932	0.98082	1	6.787	9.1325	7.8441	7.155	7.1452	10.04	6.4335	0.04815	low
GSM1607933	1.61644	0	6.6189	9.0001	8.1377	6.9954	6.9646	9.9619	6.4882	0.06293	high
GSM1607934	1.59452	0	6.8704	8.8272	7.7257	7.1365	7.1905	9.1761	6.3907	0.03391	low
GSM1607935	1.59178	0	6.7447	9.6419	7.9381	6.9292	6.6218	10.039	6.5412	0.03308	low
GSM1607936	1.51507	0	6.5455	10.0034	7.9295	6.8756	7.0603	9.5707	6.4821	0.0329	low
GSM1607937	1.5726	0	6.7076	9.0371	8.4465	7.2479	7.2325	9.9491	6.4283	0.07315	high
GSM1607938	1.65753	0	6.8017	9.1137	7.9107	6.9996	6.8802	10.3572	6.4738	0.05347	high
GSM1607939	1.65205	0	6.8901	8.8058	7.4065	7.1572	6.9408	10.2119	6.3924	0.04076	low
GSM1607940	1.59452	0	6.7279	9.0978	8.2394	6.718	7.0308	10.0702	6.4957	0.07092	high
GSM1607941	1.58082	0	6.7825	8.7526	8.1821	6.9985	7.2434	9.2263	6.4114	0.05358	high
GSM1607942	0.05479	1	6.6024	8.7692	8.2671	7.0787	7.4489	10.0107	6.4439	0.09794	high
GSM1607943	1.55068	0	6.7789	8.519	8.0904	6.5963	6.9471	9.6818	6.3526	0.07396	high
GSM1607944	1.63288	0	6.5849	9.1376	8.0483	6.9769	6.8196	9.9287	6.4585	0.05372	high
GSM1607945	1.8411	0	6.8148	8.6574	8.1473	7.2601	7.4021	9.5487	6.3875	0.06197	high
GSM1607946	1.51233	0	6.6957	8.526	8.0439	6.772	6.8059	9.6129	6.432	0.06209	high
GSM1607947	1.50411	0	6.6529	9.354	8.0194	6.9395	6.6541	9.9909	6.4563	0.0438	low
GSM1607948	1.49589	0	6.843	8.6273	8.0191	7.0861	6.9117	9.6031	6.3316	0.05096	high
GSM1607949	1.54247	0	6.7305	9.0784	7.9193	6.868	6.9825	9.9784	6.4982	0.05231	high
GSM1607950	1.70685	0	6.6832	9.6979	8.1211	6.8103	6.9476	10.0031	6.4786	0.0463	low
GSM1607951	1.63562	0	6.9279	8.9139	7.9366	6.9563	7.017	9.5285	6.393	0.0412	low
GSM1607952	0.03014	1	6.5463	9.137	7.6772	7.0013	7.1779	9.9538	6.461	0.05292	high
GSM1607953	1.58082	0	6.7961	9.1951	8.0979	6.8659	7.2641	9.3962	6.4821	0.04552	low

Supplementary Table S4. The riskscores of HNSCC patients in the GSE41613.

ID	futime	fustat	MAP2K7	MAPK3	MAPK9	ORAI1	PSMA1	UBB	ZAP70	Riskscore	Risk
GSM1020099	5.2375	0	2.35038	7.27185	7.88739	5.35665	11.4363	12.2614	3.21961	600.451	low
GSM1020100	4.63833	1	2.34295	7.12851	8.11809	4.89352	11.1529	14.3262	5.64423	678.801	low
GSM1020101	0.545	1	2.35539	6.70598	8.14898	5.57254	11.4375	14.1316	3.46897	1857.06	high
GSM1020102	0.28167	1	2.34412	6.52691	8.55935	4.77233	11.931	13.7178	2.54465	5599.54	high
GSM1020103	2.385	1	2.35981	6.67429	7.72798	4.36008	11.1661	13.9103	3.24615	2047.42	high
GSM1020104	7.08583	0	2.34328	6.18629	7.40144	4.77129	10.8935	14.063	5.60021	587.414	low
GSM1020105	3.3925	1	2.34278	6.62086	8.05243	4.7638	11.4015	13.3846	3.40725	1772.35	low
GSM1020106	7.02	0	2.35454	6.96445	8.31288	4.76942	11.4739	14.3646	3.25512	3083.19	high
GSM1020107	3.90667	1	2.35136	6.92289	7.87239	4.90809	11.147	14.5865	2.57443	3025.28	high
GSM1020108	6.91	0	2.33486	6.9337	7.9972	4.76844	10.5645	14.3109	4.576	903.486	low
GSM1020109	1.18	1	2.34956	6.86858	8.13858	4.88737	10.2359	14.0592	2.55074	1856.36	high
GSM1020110	0.8375	1	2.38393	6.29864	7.62249	5.01411	11.3313	14.3788	3.16198	2495.23	high
GSM1020111	2.37667	1	2.35481	6.94352	7.6472	4.94241	10.9747	14.264	2.56338	2049.37	high
GSM1020112	6.7925	0	2.3533	7.151	7.83519	4.14417	11.3456	13.5526	3.11991	1834.25	low
GSM1020113	0.59667	1	2.35106	6.87713	8.01849	4.30453	11.3429	14.2093	2.55507	3915.02	high
GSM1020114	0.6025	1	2.35263	6.3342	8.56091	4.35025	11.427	14.4026	2.72952	7219.58	high
GSM1020115	5.5525	1	2.35729	7.9233	7.87849	4.80341	9.95384	14.2051	2.62772	890.232	low
GSM1020116	6.52417	1	2.34844	6.91889	7.2661	4.98494	10.668	13.4416	2.54374	962.142	low
GSM1020117	0.82667	1	2.35391	6.51429	8.1977	4.88871	11.5872	14.2545	3.61329	2904.16	high
GSM1020118	1.27583	1	2.35831	6.73898	7.75617	4.22292	10.9917	14.3736	3.92834	1784.43	low
GSM1020119	6.68	0	2.35095	6.86447	7.79403	5.22822	10.7343	13.8184	3.25327	1087.67	low
GSM1020120	6.7325	0	2.35274	7.32235	7.94821	4.49243	11.0127	13.7707	3.25415	1406.49	low
GSM1020121	1.28417	1	2.35827	6.87149	8.35135	4.17813	11.4663	14.1408	3.2567	3683.34	high
GSM1020122	1.53333	1	2.34821	6.73227	7.97725	4.98016	10.6238	13.8422	3.23662	1374.08	low
GSM1020123	1.815	1	2.35857	6.69497	8.01224	4.3651	11.5106	14.1799	3.01693	3568.06	high
GSM1020124	2.02083	1	2.35629	6.7649	8.00012	4.8775	11.5611	13.9399	3.24367	2315.79	high
GSM1020125	0.4875	1	2.35856	6.7436	7.95022	3.75793	11.3899	14.0406	2.72885	4276.94	high
GSM1020126	2.95417	1	2.34903	6.92048	7.9136	5.81097	11.655	14.038	5.01171	693.199	low
GSM1020127	6.57917	0	2.37584	7.96783	7.82214	5.06737	10.9507	13.8251	2.99379	849.413	low
GSM1020128	3.43083	1	2.39167	6.88789	7.69926	5.17912	10.3904	12.947	2.559	774.169	low
GSM1020129	6.39583	1	2.34634	6.91275	7.92801	4.27012	11.0015	14.3225	3.2166	2450	high
GSM1020130	0.29333	1	2.34457	7.04712	8.6131	5.03988	11.6087	14.4765	3.84993	2735.7	high
GSM1020131	2.0725	1	2.36132	7.04891	8.27865	4.51112	10.4754	14.4366	2.68389	2672.11	high
GSM1020132	1.67583	1	2.37505	7.04128	7.93706	5.47519	10.024	13.6371	2.68909	823.214	low
GSM1020133	6.40417	0	2.35733	6.67486	7.66289	4.77809	11.0973	14.1159	3.25415	1792.3	low
GSM1020134	2.42833	1	2.35329	6.87928	7.83474	4.11122	10.6716	13.5398	4.63405	755.252	low
GSM1020135	2.23417	1	2.33789	7.68277	7.8883	4.33079	10.4941	14.175	3.06218	1273.34	low
GSM1020136	6.25583	0	2.35325	7.37104	7.24471	4.77618	10.7613	14.0989	3.22301	863.478	low
GSM1020137	3.60333	1	2.34487	6.81574	7.23461	4.76932	10.1765	14.0304	2.62373	1095.4	low
GSM1020138	6.22	0	2.34705	6.85853	8.5768	5.60277	11.1282	14.2858	3.23915	2275.29	high
GSM1020139	1.22917	1	2.35216	6.91592	8.20831	4.37629	11.3232	14.3291	3.25338	3165.4	high
GSM1020140	6.0975	0	2.34969	6.87704	7.9212	5.18591	11.383	13.9723	3.48938	1557.54	low
GSM1020141	6.02333	0	2.35278	7.00581	7.91242	5.17933	10.4758	14.3533	6.03177	348.623	low
GSM1020142	6.105	0	2.35993	6.88317	8.44581	5.46184	10.4105	14.113	3.23411	1426.17	low
GSM1020143	5.41583	1	2.34914	7.3208	7.81105	4.95976	11.4024	14.3772	3.2445	1768.08	low
GSM1020144	5.70583	0	2.34498	7.80299	8.11774	4.77814	11.0942	14.1627	3.9229	1021.42	low
GSM1020145	1.02417	1	2.33792	6.84687	7.88765	4.7471	11.9472	14.8074	3.23972	4068.69	high
GSM1020146	0.53667	1	2.35225	7.03245	8.28254	4.51648	10.9376	14.2461	4.13978	1567.5	low
GSM1020147	5.76333	0	2.35568	7.0122	7.93235	4.31585	11.2127	14.1449	4.63199	1201.42	low
GSM1020148	5.84	0	2.3319	7.05175	8.01146	4.35051	11.5325	14.2261	3.23795	2868.64	high
GSM1020149	5.84	0	2.33536	6.8374	7.70354	4.52056	10.901	14.355	2.47708	2789.2	high
GSM1020150	5.67833	0	2.34283	7.18158	7.6556	4.79917	11.2155	14.3193	3.25309	1635.07	low
GSM1020151	5.66167	0	2.35813	7.00679	7.94459	5.05283	11.4759	13.7461	2.55149	2246.26	high
GSM1020152	1.6375	1	2.35666	6.82091	8.07964	4.5495	10.568	14.2783	3.25629	1950.21	high
GSM1020153	5.62917	0	2.32856	7.37085	7.83328	5.1081	11.4168	14.0686	5.37098	541.41	low
GSM1020154	5.55	0	2.34367	7.14545	7.96777	4.62642	11.5467	14.172	3.23384	2343.71	high
GSM1020155	5.54667	0	2.34186	7.31527	7.64717	4.76208	10.5577	14.1357	3.3969	968.136	low
GSM1020156	5.435	0	2.3597	8.74609	7.9658	4.78186	10.9469	14.3237	3.25404	804.709	low
GSM1020157	0.2	1	2.35678	7.18396	7.15272	4.81212	11.5919	14.3231	2.59428	1955.75	high
GSM1020158	0.89	1	2.34283	7.2664	8.05347	5.22945	11.4115	14.2179	3.2438	1761.76	low
GSM1020159	5.45417	0	2.35034	7.13946	8.27472	3.8929	11.1259	14.1659	3.21741	3041.37	high
GSM1020160	5.295	0	2.34081	7.76828	7.66987	4.42608	10.8741	14.1907	3.99882	809.35	low
GSM1020161	5.28167	0	2.35233	7.55782	8.06066	4.81759	11.305	14.1138	4.40307	941.878	low
GSM1020162	2.92667	1	2.34316	7.37161	9.87282	4.76955	10.4956	14.316	2.53195	5416.55	high
GSM1020163	5.04583	0	2.34201	7.75516	8.04058	4.7645	10.7072	14.0891	5.41308	404.254	low
GSM1020164	5.12833	0	2.34394	6.85678	8.37973	4.45634	10.5696	14.1186	2.5152	3119.32	high
GSM1020165	1.59333	1	2.33959	6.83905	7.3693	4.54494	11.6314	14.2038	2.53041	2912.04	high
GSM1020166	5.14417	0	2.34499	6.43659	8.92804	4.73609	11.9539	14.4196	5.52466	2610.83	high
GSM1020167	4.30083	1	2.33698	6.73701	7.95553	3.89755	11.1952	14.3641	2.78973	4293.43	high
GSM1020168	5.09	0	2.36827	6.85808	7.34246	4.78379	11.6465	14.026	3.26482	1668.74	low
GSM1020169	4.95583	0	2.34066	7.15969	8.49603	4.51101	11.2474	14.3701	2.76738	3926.94	high
GSM1020170	5.08417	0	2.34734	7.13206	7.63193	4.23839	10.5332	14.3573	2.51849	2110.8	high

GSM1020171	5.05417	0	2.34	6.80414	8.3448	4.86914	11.141	14.4346	5.36146	1095.16	low
GSM1020172	4.99667	0	2.33633	6.71635	8.13801	3.86831	11.2268	14.4401	3.41964	3834.11	high
GSM1020173	0.82167	1	2.34144	7.90662	8.40442	4.191	10.5923	13.9786	3.20546	1446.83	low
GSM1020174	0.76917	1	2.29785	6.67503	8.02793	5.01526	11.1967	14.3002	3.24356	2433.75	high
GSM1020175	4.93667	0	2.35491	6.69353	7.31953	4.40703	10.6986	14.0208	2.5797	1814.16	low
GSM1020176	0.575	1	2.34723	6.72454	7.90208	4.66823	11.1347	14.3507	3.58008	2070.4	high
GSM1020177	4.8925	0	2.35205	7.8042	8.26229	4.44317	11.3381	14.0813	3.3732	1750.42	low
GSM1020178	0.10167	1	2.32645	6.44365	7.89862	4.75421	11.2847	14.4797	4.14958	2040.48	high
GSM1020179	5.2375	0	2.34763	7.3087	8.35997	4.36806	10.239	14.5369	3.24585	1903.22	high
GSM1020180	4.8025	0	2.35605	6.23687	7.92931	4.77344	11.0584	13.6658	2.5945	2782.46	high
GSM1020181	3.8275	1	2.35934	7.23409	7.72962	5.37928	10.6711	13.768	3.24793	780.29	low
GSM1020182	1.41833	1	2.36264	6.82009	7.96109	4.78147	11.2222	14.2612	3.2487	2256.57	high
GSM1020183	2.94833	1	2.36021	7.3263	7.80615	4.92214	10.6031	12.7666	3.84575	419.457	low
GSM1020184	1.20167	1	2.35647	6.3072	7.61789	5.86252	11.8885	14.6528	2.91878	3048.43	high
GSM1020185	0.545	1	2.38158	6.98801	7.96249	4.35538	11.0035	14.0385	2.56402	2687.28	high
GSM1020186	4.79417	0	2.36266	6.55835	8.29807	3.96149	11.5291	14.3792	3.23362	5259.2	high
GSM1020187	0.03833	1	2.36225	7.29587	8.14442	5.01788	11.0098	14.1581	3.24724	1567.66	low
GSM1020188	4.66833	0	2.36708	6.55232	8.42223	5.68162	11.2927	14.2724	7.12066	408.378	low
GSM1020189	4.59167	0	2.37705	7.1155	7.95526	4.63076	10.8317	14.0938	2.74935	1972.71	high
GSM1020190	1.6675	1	2.36392	7.47906	7.93035	4.44049	10.5748	13.404	4.82532	427.572	low
GSM1020191	0.69833	1	2.38031	6.99688	8.11953	4.80688	11.7165	13.8496	2.79547	2843.76	high
GSM1020192	4.5475	0	2.36178	7.47166	7.81162	4.76656	10.7373	13.9736	3.25637	1044.93	low
GSM1020193	4.53417	0	2.36737	6.88366	8.00309	4.78307	11.2348	13.9114	5.4654	677.268	low
GSM1020194	4.49	0	2.38047	7.14224	7.81417	4.74776	11.1885	13.4735	3.09066	1282.61	low
GSM1020195	4.38333	0	2.32895	6.9722	8.0545	4.69317	11.506	14.428	3.2636	2891.16	high
